# Interaction of YAP with the Myb-MuvB (MMB) complex defines a transcriptional program to promote the proliferation of cardiomyocytes

**DOI:** 10.1371/journal.pgen.1008818

**Published:** 2020-05-29

**Authors:** Marco Gründl, Susanne Walz, Laura Hauf, Melissa Schwab, Kerstin Marcela Werner, Susanne Spahr, Clemens Schulte, Hans Michael Maric, Carsten P. Ade, Stefan Gaubatz

**Affiliations:** 1 Theodor Boveri Institute and Comprehensive Cancer Center Mainfranken, Biocenter University of Wuerzburg, Wuerzburg, Germany; 2 Comprehensive Cancer Center Mainfranken, Core Unit Bioinformatics, Biocenter, University of Wuerzburg, Wuerzburg, Germany; 3 Rudolf Virchow Center for Experimental Biomedicine, University of Würzburg, Wuerzburg, Germany; Indiana University Purdue University at Indianapolis, UNITED STATES

## Abstract

The Hippo signalling pathway and its central effector YAP regulate proliferation of cardiomyocytes and growth of the heart. Using genetic models in mice we show that the increased proliferation of embryonal and postnatal cardiomyocytes due to loss of the Hippo-signaling component SAV1 depends on the Myb-MuvB (MMB) complex. Similarly, proliferation of postnatal cardiomyocytes induced by constitutive active YAP requires MMB. Genome studies revealed that YAP and MMB regulate an overlapping set of cell cycle genes in cardiomyocytes. Protein-protein interaction studies in cell lines and with recombinant proteins showed that YAP binds directly to B-MYB, a subunit of MMB, in a manner dependent on the YAP WW domains and a PPXY motif in B-MYB. Disruption of the interaction by overexpression of the YAP binding domain of B-MYB strongly inhibits the proliferation of cardiomyocytes. Our results point to MMB as a critical downstream effector of YAP in the control of cardiomyocyte proliferation.

## Author summary

YAP, the major downstream transducer of the Hippo pathway, is a potent inducer of proliferation. Here we show that the Myb-MuvB complex (MMB) mediates cardiomyocyte proliferation by YAP. We find that YAP and MMB regulate an overlapping set of pro-proliferative genes which involves binding of MMB to the promoters of these genes. We also identified a direct interaction between the B-MYB subunit of MMB and YAP. Based on the binding studies, we created a tool called MY-COMP that interferes with the association YAP to B-MYB and strongly inhibits proliferation of cardiomyocytes. Together, our data suggests that the YAP-MMB interaction is essential for division of cardiomyocytes, underscoring the functional relevance of the crosstalk between these two pathways for proper heart development.

## Introduction

The Hippo signaling pathway plays fundamental roles in proliferation and organ size control [1]. In mammals, the Hippo cascade is composed of the MST1/2 and LATS1/2 kinases and the adaptor proteins MOB1 and Salvador (SAV1). When the Hippo pathway is active, LATS1/2 phosphorylate the transcriptional coactivators YAP and TAZ, which results either in their cytoplasmic retention by 14-3-3 proteins or SCF-mediated proteasomal degradation [2]. In contrast, when Hippo is inactive, YAP/TAZ enter the nucleus and act as transcriptional coactivators predominantly by binding to DNA through TEAD transcription factors. The Hippo pathway and its downstream effectors YAP and TAZ are involved in cardiac development and have been implicated in heart regeneration after tissue damage [3,4]. Active YAP promotes proliferation of postnatal cardiomyocytes and induces a fetal-like cell state in adult cardiomyocytes [5, 6]. Similarly, cardiac-specific deletion of the Hippo kinases LATS1/2 or of the scaffold protein SAV1 results in heart enlargement and increased proliferation of embryonal and postnatal cardiomyocytes due to increased YAP activity [7,8]. Conversely, deletion of YAP suppresses cardiomyocyte proliferation [5,7,9]. Notably, activated YAP or loss of Hippo signaling can extend the neonatal proliferation of cardiomyocytes to postnatal stages, where proliferation is normally very low [5–7]. In addition, recent studies report a better outcome after myocardial infarction in mice with hyperactivated YAP [8,10–12]. Together, these studies identify YAP as an important regulator of cardiomyocyte proliferation and cardiac regeneration. However, the detailed mechanisms by which YAP promotes cardiomyocyte proliferation are still unclear [13].

We recently observed that Myb-MuvB complex (MMB) and YAP co-regulate an overlapping set of late cell cycle genes [14]. MMB consist of the evolutionary conserved MuvB core of the five proteins LIN9, LIN52, LIN54, LIN37 and RBBP4 and associated proteins [15,16]. Depending on its interactions with different binding partners, MuvB can either repress or activate transcription. Specifically, when MuvB interacts with the p130 retinoblastoma protein paralog, and with E2F4 and DP1, it forms the DREAM complex, which represses E2F-dependent gene expression in quiescent cells and in early G1 [17–19]. Upon cell cycle entry, p130, E2F4 and DP1 dissociate from the complex and the MuvB core associates with the transcription factor B-MYB to form MMB [19–22]. MMB mainly regulates genes required for mitosis and cytokinesis. Binding of YAP to enhancers promotes the activation of MMB-bound promoters, providing an explanation for the co-activation of late cell cycle genes by YAP and MMB [14].

Whether the ability of YAP to promote proliferation *in vivo* is mediated by MuvB complexes has not been addressed. To investigate the possible function of MuvB in YAP-mediated cardiomyocyte proliferation we used genetic approaches in mice and biochemical experiments. We find that YAP and MMB interact in the developing heart. *In vivo* in mice this interaction is essential for increased mitosis of Hippo-deficient cardiomyocytes. Additionally, we demonstrate that YAP driven proliferation of postmitotic cardiomyocytes is dependent on the interaction of YAP with the B-MYB subunit of MMB.

## Results

### MuvB is required for proliferation and mitosis of Hippo-deficient embryonic cardiomyocytes

**Fig 1:**
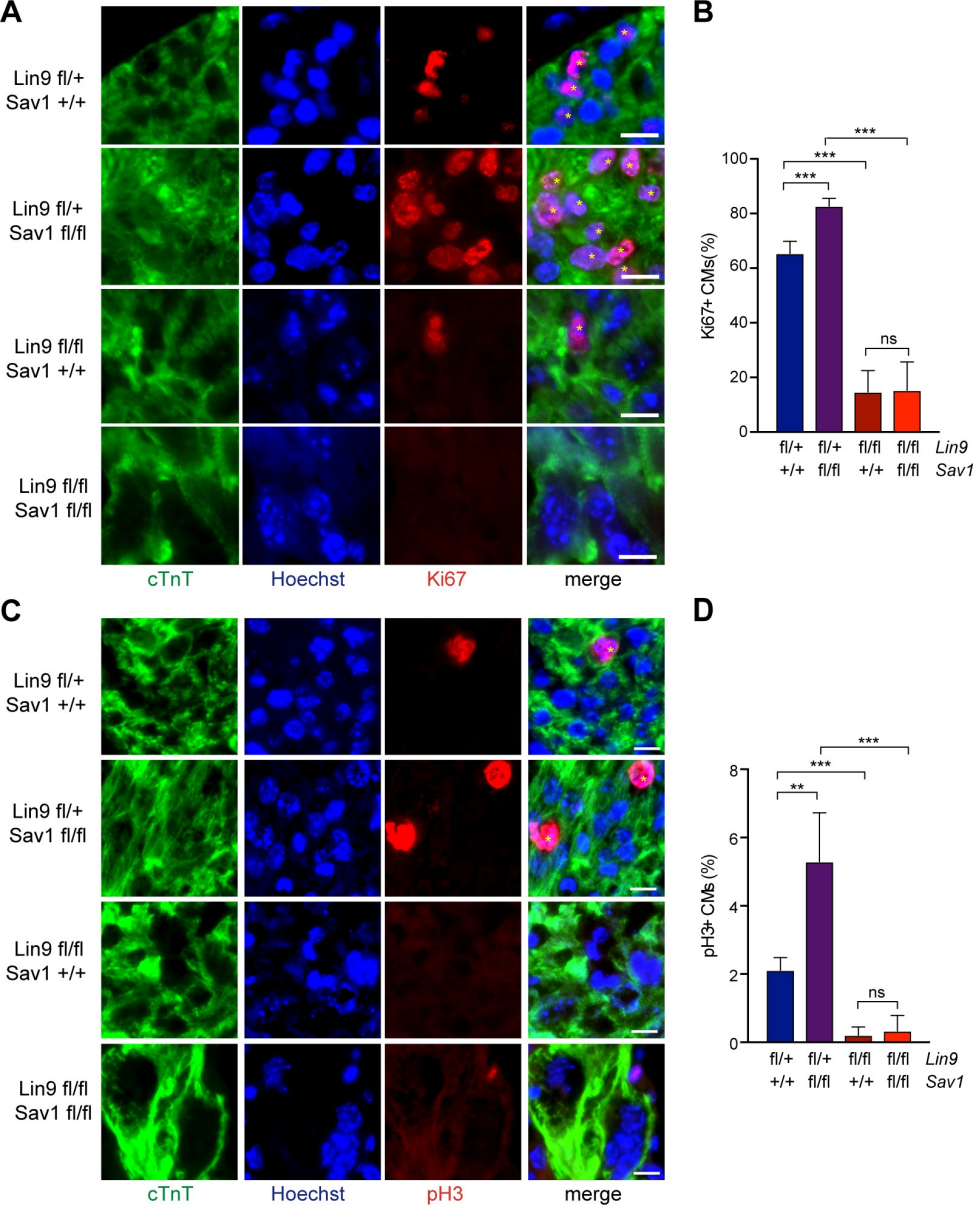
Proliferation of embryonic cardiomyocytes following Hippo inactivation depends on LIN9. A) and B) Heart sections from E13.5 mice with the indicated genotypes were stained for the proliferation marker Ki67 (red). Cardiomyocytes were identified by staining for cTnT (green). Example microphotographs are shown in (A). Asterisks indicate Ki67-positive cardiomyocyte nuclei. Scale bar: 10 μm. The quantification of Ki67-positive cells is shown in (B). Error bars indicate SDs. Number of mice analyzed per genotype: *Lin9* ^*fl/+*^*; Sav1*^*+/+*^ n = 5, *Lin9*^*fl/+*^*; Sav1*^*fl/fl*^ n = 4, *Lin9*^*fl/fl*^; *Sav1*^*+/+*^ n = 6 and *Lin9*^*fl/fl*^*; Sav1*^*fl/fl*^ n = 5 C) and D) Heart sections from E13.5 mice with the indicated genotypes were stained for the mitosis marker pH3 (red). Cardiomyocytes were identified by staining for cTnT (green). Example microphotographs are shown in (C). Asterisks indicate pH3-positive cardiomyocyte nuclei. Scale bar: 10 μm. The quantification of pH3-positive cells is shown in (D). Number of mice analyzed per genotype: *Lin9*^*fl/+*^*; Sav1*^*+/+*^ n = 5, *Lin9*^*fl/+*^*; Sav1*^*fl/fl*^ n = 4, *Lin9*^*fl/fl*^*; Sav1*^*+/+*^ n = 6 and *Lin9*^*fl/fl*^*; Sav1*^*fl/fl*^ n = 6. Error bars indicate SD. B, D: Student’s t-test. * = p<0.05, ** = p<0.01, *** = p<0.001, ns = not significant.

To explore the connection between the Hippo-YAP pathway and MuvB *in vivo* in the heart, we deleted the Hippo pathway member Salvador (*Sav1*), a scaffold protein that is required for Hippo kinase activity, and the MuvB subunit *Lin9* in cardiac precursor cells. Early cardiac specific deletion of *Lin9* and *Sav1* was achieved by using mouse strains with conditional (floxed) alleles of *Sav1* and *Lin9* in combination with Nkx2.5-Cre [23]. Phosphorylation of YAP on S127 was reduced in *Sav1* deficient hearts, indicating that YAP is hyperactivated (S1A Fig). We first examined the effect of MMB inactivation on proliferation of *Sav1* mutated cardiomyocytes. In these experiments, heterozygous *Nkx2*.*5-Cre; Lin9*^*fl/+*^ mice served as a control, because these mice developed and survived normally and showed no cardiac phenotype. To assess proliferation, we stained E13.5 heart sections for the cell cycle marker Ki67. Cardiomyocytes were identified by co-staining for cardiac troponin T (cTnT). The proportion of Ki67-positive cardiomyocytes was significantly increased in *Nkx2*.*5-Cre; Sav1*^*fl/fl*^ hearts compared to control hearts with wildtype *Sav1*, indicating that loss of SAV1 promoted cardiomyocyte proliferation as has been reported previously [7] (Fig 1A and 1B; S1B Fig). The fraction of cardiomyocytes staining positive for phosphorylated histone H3 (pH3), a marker of mitotic cells, was also increased in *Nkx2*.*5-Cre; Sav1*^*fl/fl*^ hearts (Fig 1C and 1D, S1C Fig). In contrast, proliferation and mitosis were strongly reduced by inactivation of *Lin9* and the increase in proliferation by *Sav1* deletion was blocked in *Sav1*, *Lin9* double mutant cardiomyocytes (Fig 1A–1D, S1B and S1C Fig). These genetic experiments suggest that the proliferative phenotype due to the loss of the Hippo pathway member SAV1 is dependent on the MMB subunit LIN9.

### Hippo and MMB regulate an overlapping set of genes in embryonic cardiomyocytes

**Fig 2:**
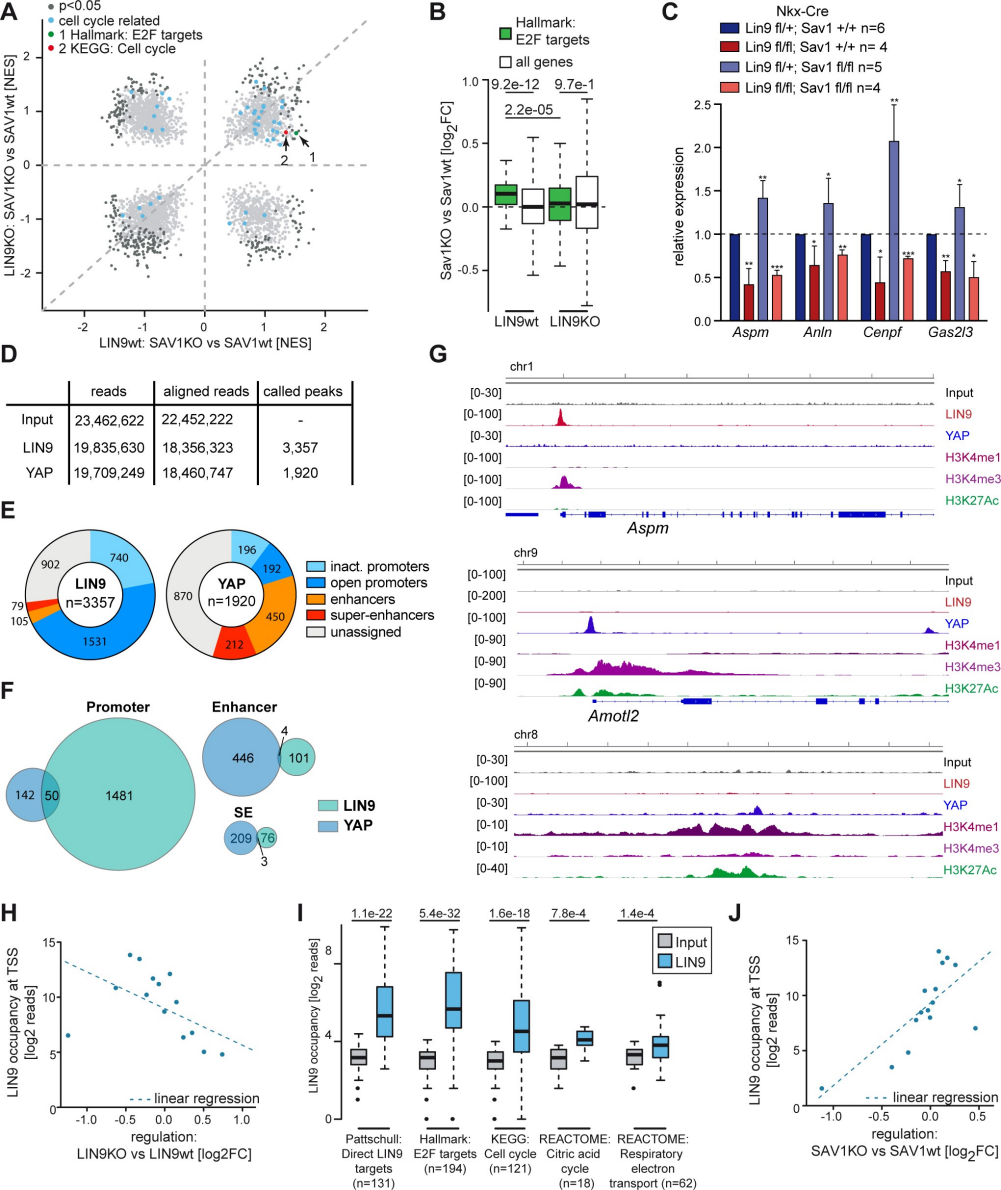
Cell cycle genes upregulated in *Sav1* knockout hearts are direct targets of LIN9. A) GSEA comparing the effect of deletion of *Sav1* in Nkx-Cre LIN9 wt and LIN9 KO heart ventricles at E13.5. Gene sets related to cell cycle are highlighted in blue. NES: normalized enrichment score. B) Boxplot comparing differences in E2F target gene expression between *Nkx2*.*5-Cre; Sav1*^*fl/fl*^ (Sav1 KO) and *Nkx2*.*5-Cre; Sav1*^*+/+*^ (Sav1 wt) heart ventricles in *Lin9*^*fl/fl*^ (LIN9 KO) or *Lin9*^*fl/+*^ (LIN9 wt) background. C) Expression of the indicated genes relative to actin and *Hprt* in heart ventricles of embryos with the indicated genotypes was analyzed by RT-qPCR. D) E16.5 hearts were subjected to ChIP-seq using antibodies specific for LIN9 and YAP. The table shows the number of reads, aligned reads and called peaks. E) Genomic localization of LIN9 and YAP in fetal (E16.5) heart ventricles. Active promoters, enhancers and super-enhancers are defined based on publicly available ChIP-seq data for histone marks. F) Venn diagram showing the overlap between binding by LIN9 and YAP in E16.5 hearts. G) Genome browser tracks illustrating the binding of LIN9 to the Aspm promoter and binding of YAP to the Amotl2 promoter and to an intergenic enhancer. ChIP-seq data for histone modifications are from ENCODE (GSE31039). Additional examples are shown in S2D Fig. H) Bin plot correlating changes in gene expression in Nkx-Cre; Lin9^fl/fl^ heart ventricles with binding of LIN9 to the promoter. Analyzed was a region 1kb upstream the TSS and input signals were subtracted. 15,642 expressed genes were grouped into 15 bins and the mean of each bin is plotted. Dashed line: Regression based on a linear model. I) Boxplot depicting LIN9 binding to genes from the gene sets shown in S2A and S2D Fig. Analyzed was a region of +/-250bp around the TSS. J) Bin plot correlating changes in gene expression after *Sav1* knockout in *Nkx2*.*5-Cre* hearts with binding of LIN9 to the promoters at E16.5. Analysis was performed as in H.

Next, we explored the impact of *Lin9* mutation on the transcriptional program of Hippo-deficient cardiomyocytes by performing RNA-seq using RNA isolated from heart ventricles of E13.5 embryos carrying cardiac specific deletions of *Sav1* alone, *Lin9* alone or *Sav1* and *Lin9* together. In *Sav1* knockout hearts, E2F target genes and other gene sets related to cell cycle regulation were enriched after inactivation of Hippo-signaling (Fig 2A, S2A and S2B Fig). Strikingly, the activation of these gene sets was suppressed by the simultaneous deletion of *Lin9* together with *Sav1*, indicating that the elevated expression of these genes in *Sav1* mutant hearts requires the function of MMB (Fig 2A and 2B, S2A and S2B Fig). By RT-qPCR we independently validated that a set of cell cycle target genes were activated in *Sav1* mutated hearts, downregulated in *Lin9* mutated hearts and remained downregulated in hearts isolated from double mutant embryos (Fig 2C).

To find out whether cell cycle genes upregulated in *Sav1* hearts are direct targets of MMB, we performed chromatin immunoprecipitation experiments followed by high-throughput sequencing (ChIP-seq) (Fig 2D). We identified 3,357 binding sites for LIN9 in the fetal heart. By comparison with previously reported ChIP-seq data of histone modifications characteristic for active promoters and enhancers, most LIN9 binding sites are located in active promoters while less than 5% of LIN9 sites are found in enhancers or super-enhancers (Fig 2E, S2C Fig). ChIP-seq of YAP in E16.5 hearts showed that YAP predominantly binds to enhancers and super-enhancers rather than to promoters (Fig 2E), which is consistent with recent genome wide studies in human cell lines [14,24–26]. Consequently, there was little overlap in the binding of YAP and LIN9 (Fig 2F, S2C Fig). Genome browser tracks of LIN9 and YAP-bound promoters and of YAP-bound enhancers illustrating these findings are shown in Fig 2G and S2D Fig.

To identify the direct targets of LIN9 in the heart, we plotted changes in gene expression upon deletion of *Lin9* against the density of promoter-bound LIN9. This revealed a correlation between genes that are activated by LIN9 (i.e. that are downregulated after deletion of *Lin9*) and promoter binding of LIN9 (Fig 2H). In particular, LIN9 strongly bound to the promoters of cell cycle genes and E2F target genes (Fig 2I). While overall LIN9-binding correlated with expression changes, LIN9 only weakly bound to the promoters of some gene sets that are also downregulated in *Lin9* knockout hearts, including genes related to mitochondrial function, oxidative phosphorylation, metabolism, heart muscle contraction and ion channel activity (Fig 2I, S2E and S2F Fig). Downregulation of these genes is likely an indirect consequence of loss of *Lin9*. Strikingly, plotting changes in gene expression upon cardiac-specific deletion of *Sav1* against LIN9-promoter occupancy showed that promoters of genes activated in *Sav1* knockout hearts were bound by LIN9, indicating that they are direct targets of MMB (Fig 2J). Altogether these data support the idea that YAP activates LIN9-bound cell cycle promoters from distant enhancers as previously shown in human MCF10A cells [14]. In summary, the deletion of *Sav1* hyperactivates YAP, which in turn results in induction of cell cycle genes whose promoters are bound by LIN9.

### Lin9 is required for proliferation of Hippo-deficient postnatal cardiomyocytes

**Fig 3:**
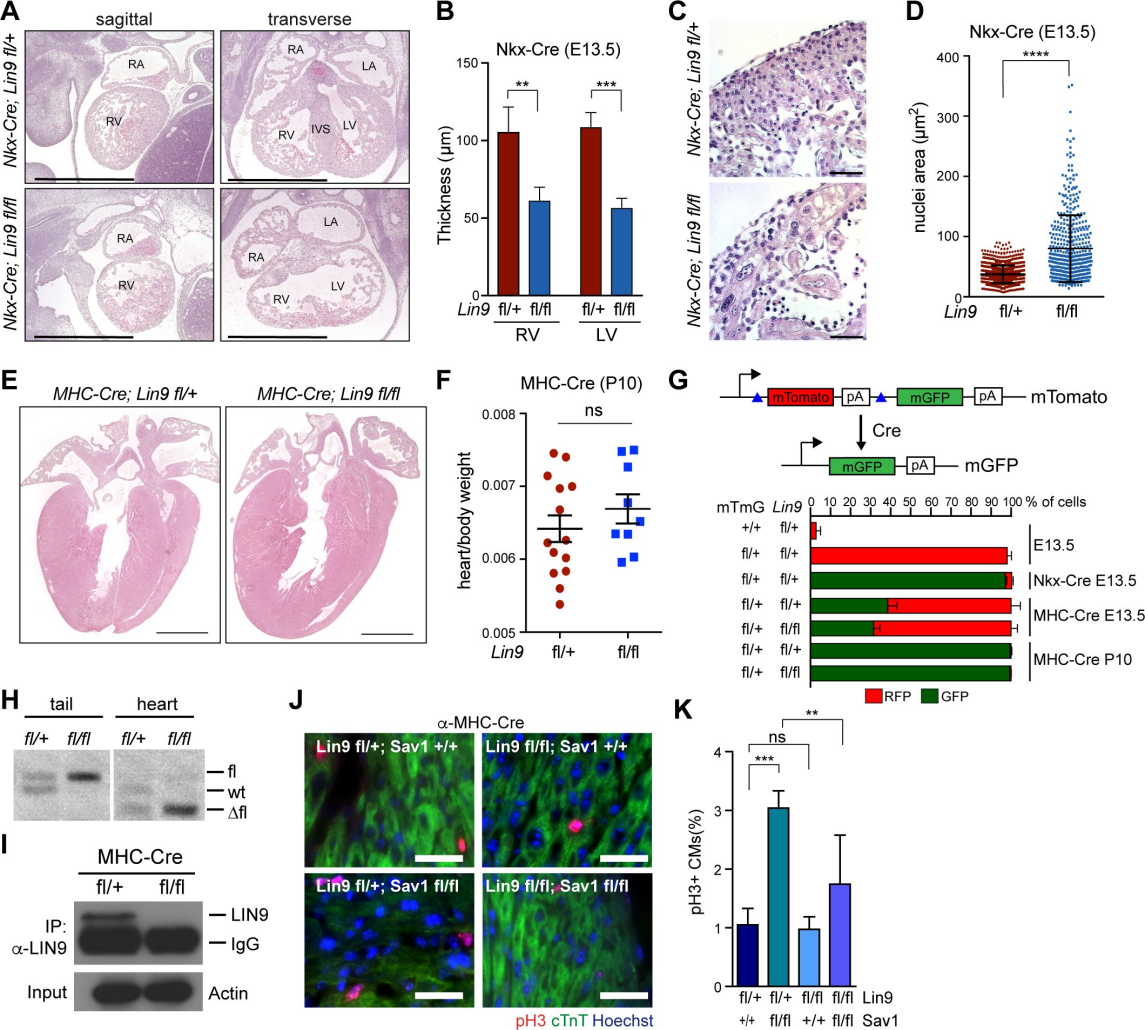
LIN9 is required for cardiomyocyte proliferation in Hippo-deficient postnatal hearts. A) H&E-stained sagittal and transverse sections of embryonic E13.5 hearts of mice with the indicated genotypes. RA: right atrium, RV: right ventricle, LA: left atrium, LV: left ventricle, IVS: interventricular septum. Scale bar: 1 mm B) Thickness of the right and left ventricular compact myocardium. n = 5 *Lin9*^*fl/+*^ hearts and n = 3 *Lin9*^*fl/fl*^ hearts. C) High magnification of the myocardium of *Lin9*^*fl/fl*^ embryonic hearts demonstrating pleomorphic and enlarged nuclei. Scale bar: 25μm D) Quantification of the nuclear area in the myocardium of *Lin9*^*fl/+*^ and *Lin9*^*fl/fl*^ embryonic hearts. E) H&E-stained sections of hearts from *α-MHC-Cre; Lin9*^*fl/+*^ and *α-MHC-Cre; Lin9*^*fl/fl*^ mice at P10. Scale bar: 1 mm F) heart to body weight of *α-MHC-Cre; Lin9*^*fl/+*^ and *α-MHC-Cre; Lin9*^*fl/fl*^ mice at P10. n = 14 control mice and n = 9 *Lin9*^*fl/fl*^ mice. G) Top: Scheme of the mT/mG reporter. Upon expression of Cre-recombinase, mTomato is deleted and mGFP is activated. Bottom: Quantification of mTomato (mT) and mEGFP (mG) positive cardiomyoyctes. n = 3 to 4 mice per genotype. Example FACS data are shown in S3D Fig. H) PCR genotyping using primers to detect the wt, fl and Δfl allele of *Lin9* demonstrating efficient deletion of *Lin9* in the hearts of 10 days old *α-MHC-Cre; Lin9*^*fl/fl*^ mice. Genomic DNA extracted from tail biopsies was used as control. I) To confirm that the LIN9 protein is lost in *α-MHC-Cre; Lin9*^*fl/fl*^ hearts, lysates from P10 hearts with the indicated genotypes were immunoprecipitated and immunoblotted with anti-LIN9 antibodies. Immunoblotting of lysates with an Actin antibody served as a control. IgG denotes the heavy chain of the immunoprecipitating primary antibody. J) and K) Fraction of pH3-positive cardiomyocytes in the hearts of 10 days old mice with the indicated genotypes. Cardiomyocytes were identified by staining for cTnT. Scale bar 25μm. *Lin9*^*fl/+*^; *Sav1*^*+/+*^ n = 3, *Lin9*^*fl/+*^; *Sav1*^*fl/fl*^ n = 6, *Lin9*^*fl/fl*^*;Sav1*^*+/+*^ n = 3 and *Lin9f*^*l/fl*^*;Sav1*^*fl/fl*^ n = 7. B),D), I): Error bars indicate SDs. Error bars indicate SEM. Student’s t-test. * = p<0.05, ** = p<0.01, *** = p<0.001, ns = not significant.

Deletion of *Lin9* in heart progenitor cells by Nkx2.5-Cre resulted in embryonic lethality due to defects in cardiomyocyte proliferation and division resulting in enlarged nuclei and leading to reduced thickness of the compact myocardium of both ventricles (Fig 3A–3D, S3A Fig). The *Nkx2*.*5-Cre; Lin9*^*fl/fl*^*; Sav*^*fl/fl*^ double knockout mice phenocopied the morphological defects of *Nkx2*.*5-Cre; Lin9*^*fl/f*l^ single mutants (S3B Fig). This is consistent with the reduced expression of cell cycle genes in the hearts of *Nkx2*.*5-Cre; Lin9*^*fl/fl*^ and *Nkx2*.*5-Cre; Lin9*^*fl/fl*^*; Sav*^*fl/fl*^ double knockout mice.

To circumvent the embryonic lethality associated with deleting *Lin9* in early cardiac precursors, we used a transgenic mouse line with Cre recombinase driven by the alpha-MHC-promoter, which is active at a later stage during heart development compared to *Nkx2*.*5-Cre* [27]. *α-MHC-Cre; Lin9*^*fl/fl*^ mice survived into adulthood without any obvious heart phenotype and without differences in the heart to body weight when compared to heterozygous control animals (Fig 3E and 3F, S3C Fig). To investigate the efficiency of Cre mediated deletion in the heart, we used a *mT/mG* reporter strain [28]. Upon Cre-induced recombination of the *mT/mG* reporter gene, membrane bound Tomato (mT) is removed and the expression of membrane bound EGFP (mG) is activated (Fig 3G, S3D Fig). In control mice that do not harbor any Cre recombinase, no recombination of the *mT/mG* reporter gene took place and more than 95% of cardiomyocytes exhibited red fluorescence, as expected. After combining *Nkx2*.*5-Cre* with the *mT/mG* reporter strain more than 95% of cardiomyocytes exhibited green fluorescence in 13.5 dpc hearts, indicating efficient recombination. In contrast, in *α-MHC-Cre; mT/mG* mice, a much smaller proportion of about 30–40% cardiomyocytes were GFP positive at E13.5. The relative low recombination frequency in *α-MHC-Cre* mice at this developmental time point can explain the lack of an embryonic heart phenotype in *α-MHC-Cre*; *Lin9*^*fl/fl*^ mice. However, at P10, recombination in the heart of *α-MHC-Cre* mice was almost 100%. Cardiac specific deletion of *Lin9* in *α-MHC-Cre* mice at P10 was confirmed by genomic PCR and, importantly, the absence of the LIN9 protein was validated by IP-western (Fig 3H and 3I). Taken together while early cardiac deletion of *Lin9* caused a dramatic and embryonal lethal phenotype, postnatal deletion by *α-MHC-Cre* did not result in a heart phenotype.

In hearts of 10 days old wildtype mice, LIN9 remained associated with chromatin as determined by ChIP-seq (S3E–S3G Fig). The overall chromatin binding pattern of LIN9 was similar in embryonic and postnatal hearts and the shared binding sites reflect high confident LIN9-binding sites (S3G–S3J Fig).

To address whether LIN9 is required for mitosis of postnatal cardiomyocytes in Hippo-deficient hearts, we next crossed *α*-*MHC-Cre; Lin9*^*fl/fl*^ mice to *Sav1*^*fl/fl*^ mice. Under normal conditions, there are almost no dividing cardiomyocytes in the postnatal heart, as expected. The deletion of *Sav1* robustly increased the fraction of mitotic cardiomyocytes (Fig 3J and 3K). Strikingly, this phenotype was suppressed when *Lin9* was lacking and the fraction of mitotic cardiomyocytes remained low in *α-MHC-Cre*; *Sav1*^*fl/fl*^*; Lin9*^*fl/fl*^ double mutant mice, indicating a role for MMB in mitotic entry of postnatal cardiomyocytes due to Hippo deficiency (Fig 3J and 3K).

Thus, LIN9 is dispensable in the postnatal heart probably because cardiomyocytes hardly divide at this time point. However, LIN9 becomes necessary for ectopic cardiomyocyte proliferation in the absence of SAV1.

### Cardiomyocyte proliferation by activated YAP requires LIN9

**Fig 4:**
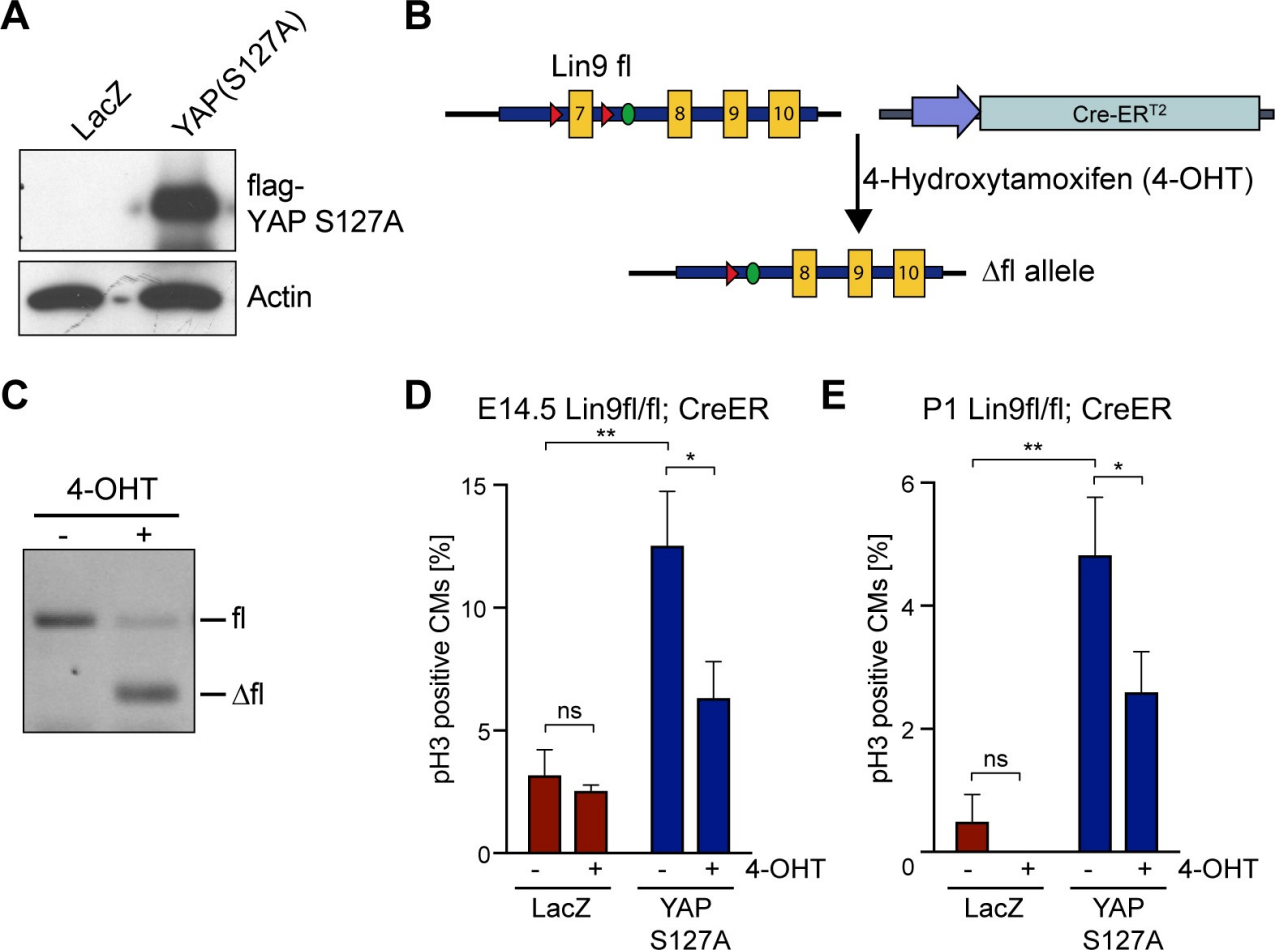
Cardiomyocyte proliferation by activated YAP requires MMB. A) Cardiomyocytes isolated from neonatal (P1) *Lin9*^*fl/fl*^; *CreER*^*T2*^ mice were transduced with a LacZ control (Ade-LacZ) or FLAG-YAP(S127A) expressing adenovirus. Expression of YAPS127A was verified by immunoblotting with a flag-antibody 72 hours after adenoviral transduction. Actin served as a control. B) Scheme of the floxed Lin9 locus and the *CreER*^*T2*^ transgene. After addition of 4-OHT, *CreER*^*T2*^ is activated to delete the floxed exon 7 of Lin9 [37]. Lox sites are indicated by red triangles. C) Cardiomyocytes isolated from *Lin9*^*fl/fl*^; *CreER*^*T2*^ hearts were treated without and with 4-OHT as indicated and the deletion of *Lin9* was verified by genomic PCR. D) and E) Embryonal (E14.5) *Lin9*^*fl/fl*^; *CreER*^*T2*^ cardiomyocytes (D) or postnatal (P1) cardiomyocytes (E) were transduced with Ade-LacZ or with Ade-YAP[S127A] and treated with or without 4-OHT. The fraction of pH3-positive cardiomyocytes was quantified. Example microphotographs are shown in S4 Fig. Error bars show SDs of biological replicates (n = 3).

To directly test whether increased cardiomyocyte proliferation due to activated YAP depends on MMB, we transduced neonatal cardiomyocytes with an adenovirus expressing a constitutive active version of YAP in which S127, the site of the inactivating phosphorylation by LATS kinases, is mutated to alanine (Fig 4A). To determine the requirement of MMB for YAP(S127A) induced proliferation, we used cardiomyocytes isolated from mice with a conditional allele of *Lin9* (*Lin9*^*fl/fl*^) and expressing a hormone inducible CreER recombinase that can be activated by the addition of 4-hydroxytamoxifen (4-OHT) (Fig 4B). Treatment with 4-OHT resulted in efficient deletion of *Lin9* in neonatal *Lin9*^*fl/fl*^; *CreER* cardiomyocytes (Fig 4C). Expression of YAP(S127A) robustly induced mitotic entry of embryonic E14.5 and postnatal P1 cardiomyocytes, as reported before [5,9] (Fig 4D and 4E, S4A and S4B Fig). Importantly, when *Lin9* was deleted by treatment with 4-OHT, the increase in pH3 positive cells by YAP(S127A) was strongly suppressed, indicating that YAP requires MMB to promote cardiomyocyte proliferation (Fig 4D and 4E, S4A and S4B Fig).

### YAP physically interacts with MMB in cardiomyocytes

**Fig 5:**
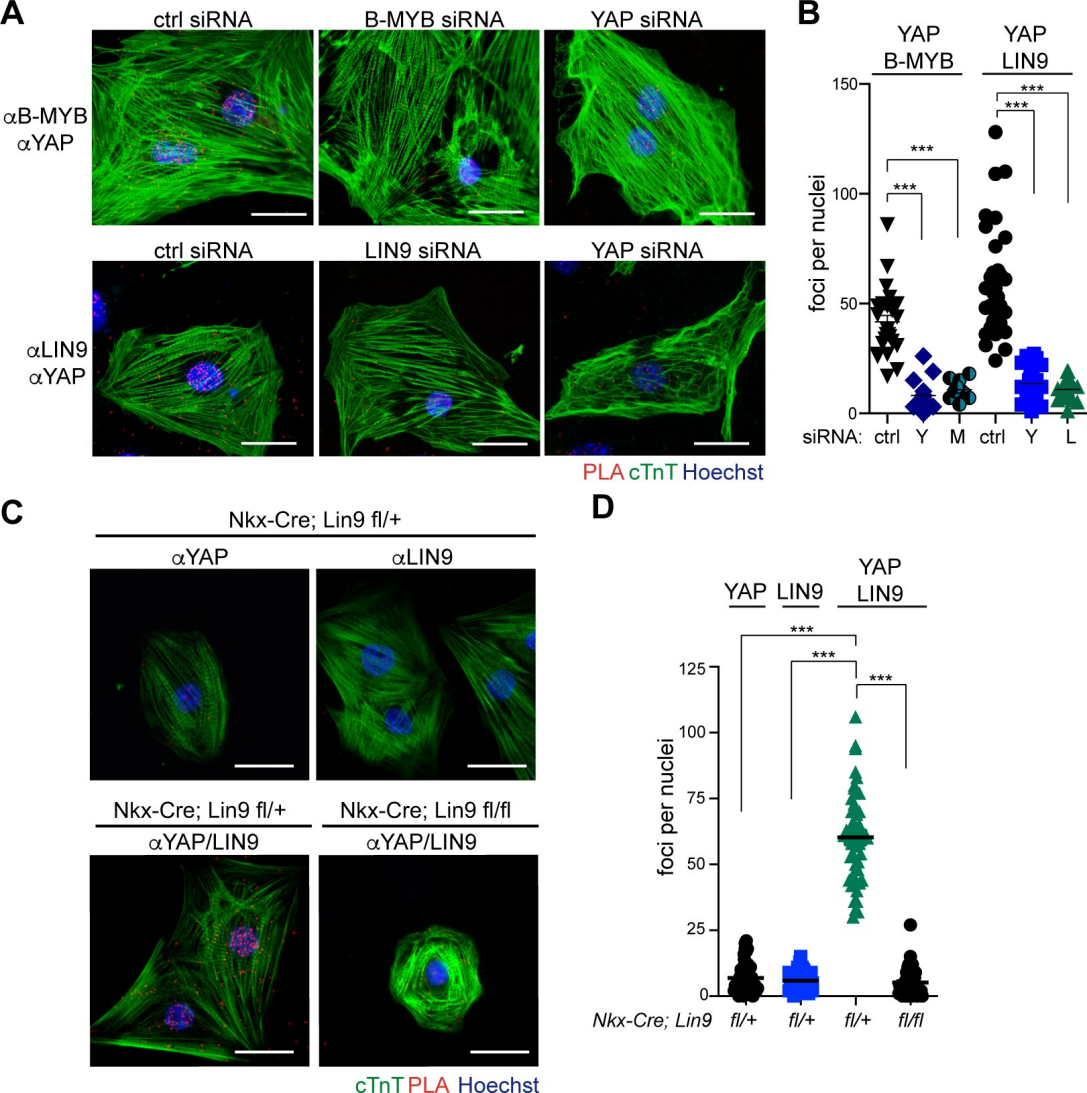
Interaction between YAP and MMB in cardiomyocytes. A) Proximity ligation assays (PLA) showing that YAP and LIN9 and YAP and B-MYB interact in the nuclei of E14.5 cardiomyocytes. siRNA mediated depletion of YAP/TAZ, LIN9 or B-MYB served as control. Interactions are indicated by red fluorescent dots. Cardiomyocytes were identified by immunostaining for cTnT (green). Bar: 25 μm B) PLA shown in A were quantified by counting the number of interactions per nucleus. C) PLA of YAP and LIN9 showing that the signal is lost when Lin9 is deleted in *Nkx2*.*5-Cre*, *Lin9*^*fl/fl*^ mice. Incubation with either YAP or LIN9 antibody served as an additional negative control. Cardiomyocytes were identified by immunostaining for cTnT (green). Bar: 25 μm. D) Quantification of the PLA shown in C.

YAP and MMB could independently regulate a similar set of genes required for cell cycle regulation or they could cooperate by binding to each other. To address these possibilities, we next investigated whether YAP and B-MYB interact in the heart. Proximity ligation assays (PLA) showed that YAP indeed interacted with B-MYB in embryonic cardiomyocytes (Fig 5A and 5B). The interaction was specific, as demonstrated by the loss of the PLA signal upon siRNA-mediated depletion of B-MYB or YAP (Fig 5A and 5B). YAP also interacted with LIN9, a core subunit of the MuvB complex that is required for binding of B-MYB to MuvB [29]. Binding between YAP and LIN9 was specific as demonstrated by siRNA mediated depletion of LIN9 or YAP (Fig 5A and 5B). Furthermore, the PLA signal was lost when one of the two antibodies was omitted or after genetic deletion of *Lin9* in *Nkx2*.*5-Cre; Lin9*^*fl/fl*^ cardiomyocytes, providing further evidence for an interaction between YAP and MMB in cardiomyocytes (Fig 5C and 5D).

### B-MYB interacts with the tandem WW domains of YAP

**Fig 6:**
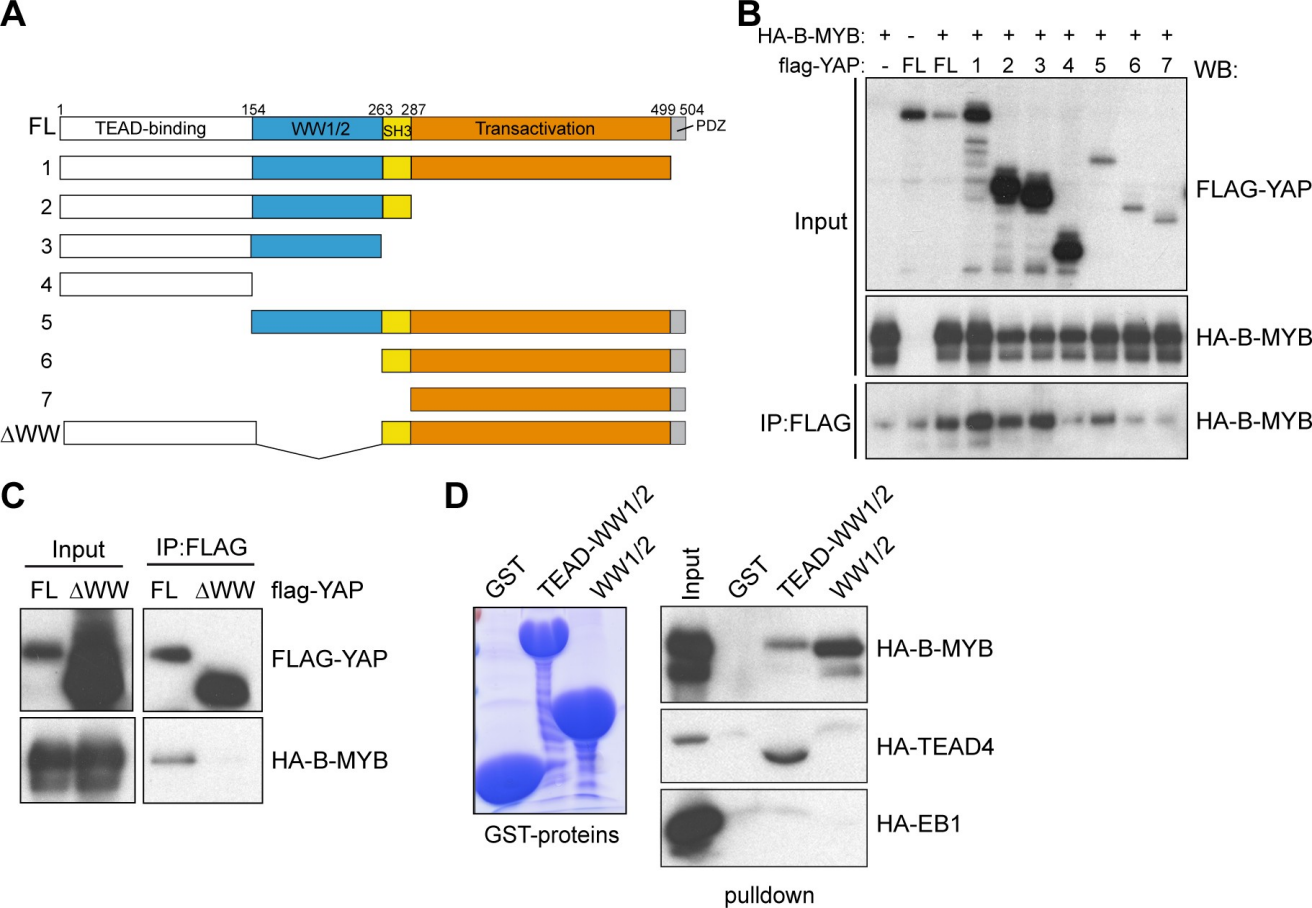
The WW domains of YAP mediate the interaction with B-MYB. A) Scheme of YAP deletion mutants used in co-immunoprecipitation experiments. B) Co-immunoprecipitation of ectopically expressed HA-B-MYB with flag-tagged YAP. Lysates of HeLa cells expressing HA-B-MYB and flag-YAP mutants (see A) were immunoprecipitated with flag-antiserum and immunoblotted with an anti-HA-antibody. 3% percent of total lysate was immunoblotted (Input). Background bands of faster mobility than the full-size proteins are most likely degradation products. A quantification of the experiment is provided in S5A Fig. C) Co-immunoprecipitation between flag-YAP and HA-B-MYB demonstrating that the WW domains of YAP are required for the interaction with B-MYB. D) Pulldown experiments with 5 μg of the indicated recombinant GST-fusion proteins and with HA-B-MYB, HA-TEAD4 and HA-EB1 expressed in HeLa cells. Bound proteins were detected with an anti-HA antibody. HA-TEAD was used as a positive control for the interaction with GST-YAP-TEAD-WW1/2 and HA-EB1 served as a negative control. A coomassie brilliant blue stained gel of the purified proteins is shown to the left.

To gain further insights into the interplay between YAP and B-MYB and to develop tools to interfere with the interaction, we mapped the domains of YAP and B-MYB that are involved in the interaction. In co-immunoprecipitation experiments with HA-B-MYB and a set of truncated flag-tagged YAP constructs, B-MYB interacted with YAP only when the tandem WW domains were present. B-MYB did not interact with the C-terminal transactivation domain or the PDZ-binding motif of YAP (Fig 6A and 6B, S5A Fig). Internal deletion of the tandem WW domains abolished the binding, confirming that these domains are required for the YAP-B-MYB interaction (Fig 6C). To verify that the YAP WW domains mediate the interaction with B-MYB, we performed pulldown experiments with recombinant GST fused to the N-terminal part of YAP containing the TEAD-binding and WW domains (GST-TEAD-WW1/2) or just the two WW domains (GST-WW1/2) (S5B Fig). HA-B-MYB specifically bound to GST-TEAD-WW1/2 and GST-WW1/2 and but not to GST alone (Fig 6D). As a control, HA-TEAD4 only bound to GST-TEAD-WW1/2 containing the TEAD-binding domain, but not to GST alone or to GST-WW1/2. HA-tagged EB1, which was used as a negative control, did not bind to any of the GST constructs. Although B-MYB can independently bind to both WW domains, it more strongly interacted with the first WW domain and strongest binding of B-MYB was observed when both WW domains were present (S5C Fig).

### Overexpression of the YAP binding domain disrupts the B-MYB-YAP interaction and inhibits cardiomyocyte proliferation

**Fig 7:**
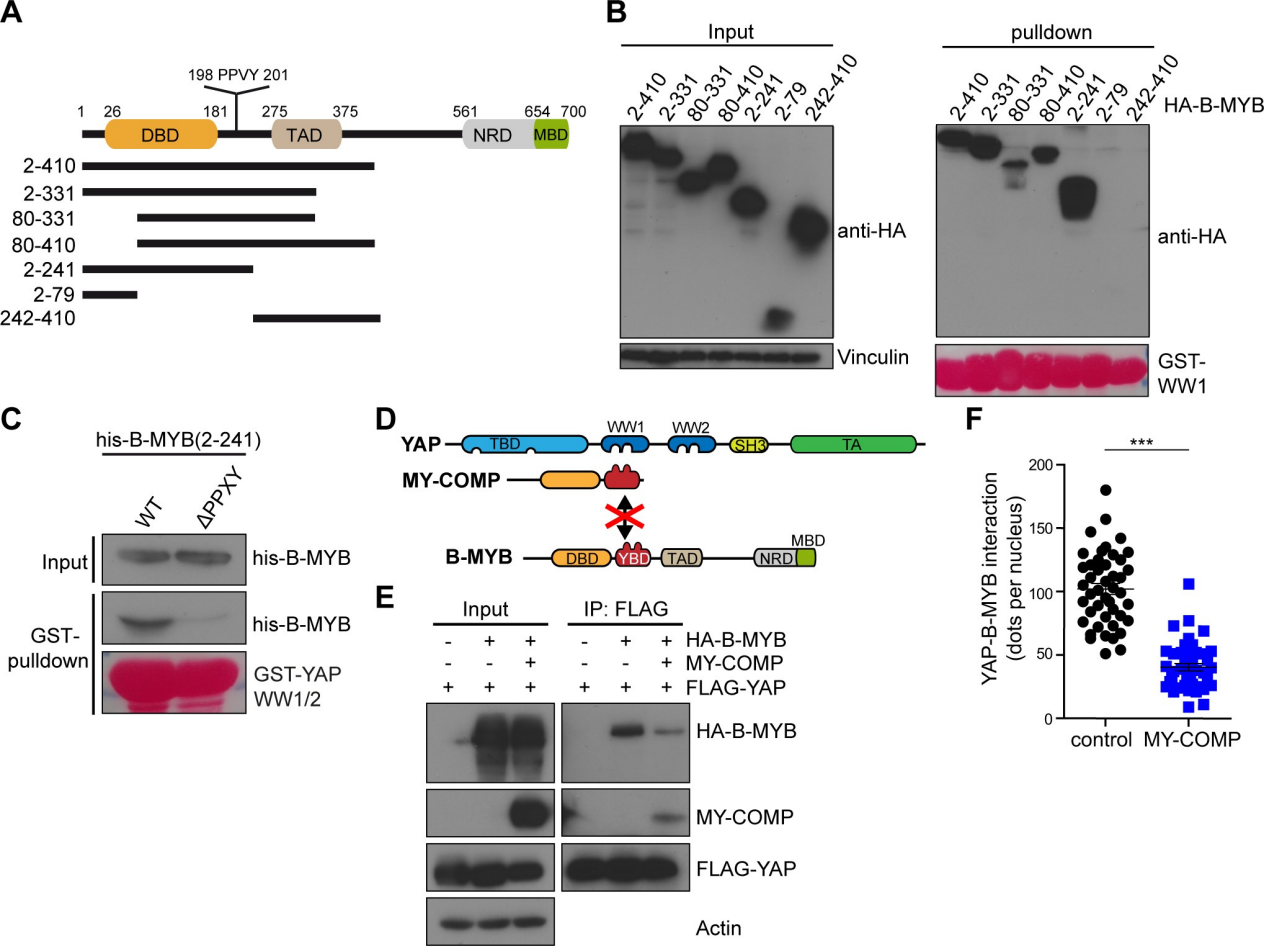
Disrupting the association between B-MYB and YAP by MY-COMP. A) Scheme of the domain structure of B-MYB and deletion constructs. B) Pulldown experiments with GST-WW1 and HA-tagged B-MYB constructs. Bound proteins were detected by immunoblotting with a HA antibody. Input: 3% of the lysate. Vinculin was used as a control. Recombinant GST-proteins were detected by Ponceau staining. C) Pulldown experiments with GST-WW and his-tagged B-MYB(2–241) and B-MYB(2–241,ΔPPXY). Bound B-MYB was detected with a his-antibody. Input: 3% of the recombinant his-tagged B-MYB constructs. Recombinant proteins were detected by Ponceau S staining. D) Scheme for the disruption of the YAP-B-MYB interaction by MY-COMP. E) HeLa cells were transfected with flag-YAP, HA-B-MYB and MY-COMP (HA-NLS-B-MYB(2–241)) as indicated. Flag-YAP was immunoprecipitation and bound HA-B-MYB was detected by immunoblotting. When MY-COMP was co-transfected, the interaction between flag-YAP and HA-B-MYB was decreased. Input: 3% of the lysate. A quantification is shown in Suppl. S6C Fig. F) PLA of endogenous YAP and B-MYB upon transfection of MY-COMP showing that the MY-COMP disrupts the interaction between YAP and B-MYB. Example microphotographs are shown in S6D Fig. Error bars indicate SEMs. Student’s t-test. *** = p<0.001.

Since YAP binds to the N-terminus of B-MYB and since the MMB-interaction domain (MBD) is located in the C-terminal region, the binding of YAP to B-MYB is likely not mediated by binding of YAP to the MuvB core [14,29]. In pulldown experiments with GST-WW1 and with a set of HA-tagged B-MYB deletion mutants we mapped the minimal binding site for YAP to amino acids 80 and 241 of B-MYB (Fig 7A and 7B). For example, B-MYB(2–241) robustly bound to YAP whereas B-MYB(2–79) and B-MYB(242–410) failed to bind to YAP (Fig 7B). The interaction between B-MYB and YAP could be indirect since exogenously expressed HA-B-MYB likely associates with additional cellular proteins. Purified, recombinant his-tagged B-MYB(2–241) interacted with GST-WW1/2, indicating that the binding between B-MYB and YAP is direct (Fig 7C).

To identify the region in B-MYB that is responsible for binding to the WW domains, we used an overlapping peptide library in μSPOT format that displayed the whole length of the B-MYB protein as 15mer peptides with 2 amino acid offset. The peptide array was incubated with GST-WW1/2 and binding was detected with an anti-GST antibody. These experiments confirmed direct binding of B-MYB to the WW domains of YAP and identified a 15 amino acid peptide containing a PPXY motif as the fragment with the highest YAP binding capacity (S6A and S6B Fig). Proline rich PPXY motifs are known ligands for the WW domain, a protein-interaction domain characterized by two tryptophan-residues separated by 20 to 22 amino acids [30,31]. To directly test whether the PPXY motif is required for binding to YAP, we performed pulldown experiments with a deletion mutant of recombinant B-MYB lacking the PPXY sequence (ΔPPXY). Compared to his-tagged B-MYB(2–241), binding of his-B-MYB(2–241,ΔPPXY) to the WW domains of YAP was strongly reduced (Fig 7C). The PPXY motif was also required for the interaction with YAP in the context of the full-length B-MYB protein in cells (S6C and S6D Fig). Taken together, these results indicate that the YAP interacting region of B-MYB is located between amino acids 80 and 241 of B-MYB and involves a PPXY motif.

Since B-MYB and YAP directly interact, we next asked whether overexpression of the YAP binding domain of B-MYB can interfere with the interaction of B-MYB with YAP due to direct competition for the binding site (Fig 7D). To address this possibility we created B-MYB(2–241) fused to a HA tag and a nuclear localization signal (HA-NLS-B-MYB-2-241), and named it MY-COMP for MYB-YAP competition. First, we expressed MY-COMP in cells and performed co-immunoprecipitation experiments. In HeLa cells that express MY-COMP, the amount of full-length HA-B-MYB co-precipitating with flag-YAP was strongly reduced when compared to control transfected cells (Fig 7E, S6E Fig). Expression of MY-COMP also interfered with the endogenous YAP and B-MYB interaction as determined by proximity ligation assays (PLA) (Fig 7F, S6F Fig).

**Fig 8:**
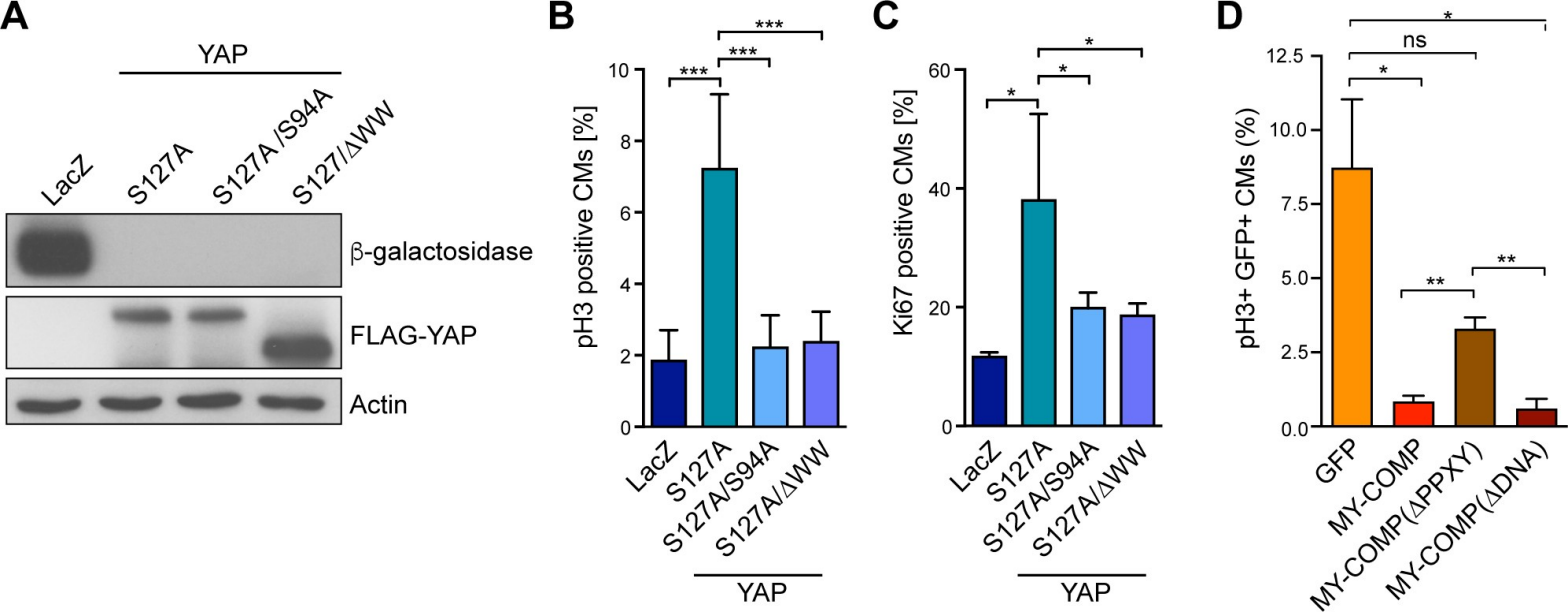
The YAP-B-MYB interaction is required for cardiomyocyte proliferation. A) Cardiomyocytes isolated from P1 hearts were transduced with Ade-LacZ, Ade-YAP[S127A], and Ade-YAP[S127A/S94A] or with Ade-YAP[S127A/ΔWW]. Expression of ß-galactosidase and of flag-tagged YAP constructs was verified by immunoblotting. Actin served as a loading control. B) and C) Cardiomyocytes were transduced with the constructs as described in A. The fraction of pH3-positive positive (B) and Ki67-positive (C) cardiomyocytes was determined. Example microphotographs are shown in S7A and S7B Fig. Error bars show SD. (B) n = 6–8, (C) n = 3–4 biological replicates. D) Disruption of the YAP-B-MYB interaction by MY-COMP inhibits mitosis of cardiomyocytes. Embryonal cardiomyocytes were infected with adenoviruses expressing GFP, MY-COMP, MY-COMP, ΔPPXY or with MYCOMP, ΔDNA each coupled to GFP through a T2A self-cleaving peptide. Infected cardiomyocytes were detected by staining for cTnT and by their green fluorescence. Mitotic cells were quantified by staining for phospho-H3. Example microphotographs are provided in S7C Fig. Error bars indicate SEMs. Student’s t-test. * = p<0.05, ** = p<0.01, *** = p<0.001, ns = not significant.

Next, we asked whether the interaction between YAP and B-MYB is important for promoting proliferation in neonatal cardiomyocytes. We first tested whether the ability of YAP[S127A] to stimulate neonatal cardiomyocyte proliferation and to promote entry into mitosis depends on the WW domains, which mediate the binding to MMB. To do so, we transduced P1 cardiomyocytes with adenoviral constructs encoding for LacZ or YAP[S127A] (Fig 8A) and measured induction of mitosis and proliferation by phospho-H3 and Ki67 staining. While YAP[S127A] robustly induced mitosis and proliferation, YAP[S127A] lacking the WW domains (YAP[S127A/ΔWW]) failed to increase the fraction of pH3 and Ki67-positive cardiomyocytes (Fig 8B and 8C, S7A and S7B Fig). Similarly, a point mutant of YAPS127A deficient in binding to TEAD (YAP[S127A/S94A]), was also not able to increase proliferation and mitosis, indicating that these functions of YAP are TEAD dependent (Fig 8A–8C, S7A and S7B Fig).

We next asked whether the interaction between YAP and B-MYB is critical for the high proliferation rate of embryonal cardiomyocytes. To address this question, we infected embryonal cardiomyocytes with an adenovirus expressing MY-COMP coupled to GFP through a T2A self-cleaving peptide. Infected cardiomyocytes were detected by their green fluorescence and by staining for cTnT (S7C Fig). Strikingly, staining for phospho-H3 showed that expression of MY-COMP strongly suppressed mitosis of embryonal cardiomyocytes (Fig 8D, S7C Fig). Importantly, this effect was diminished by deletion of the PPXY motif in MY-COMP, indicating that the ability to prevent proliferation correlates with the ability to disrupt the YAP-B-MYB interaction. The effect of MY-COMP is not due to interference with the DNA-binding of B-MYB, because expression of MY-COMP with a mutation in the DNA-binding domain that has been shown to prevent the interaction with DNA [32], showed the same phenotype. Taken together these observations are consistent with the notion that the YAP-dependent neonatal cardiomyocyte proliferation is mediated by the interaction of YAP with MMB.

### MMB target genes are downregulated in differentiated cells and re-activated by YAP

**Fig 9:**
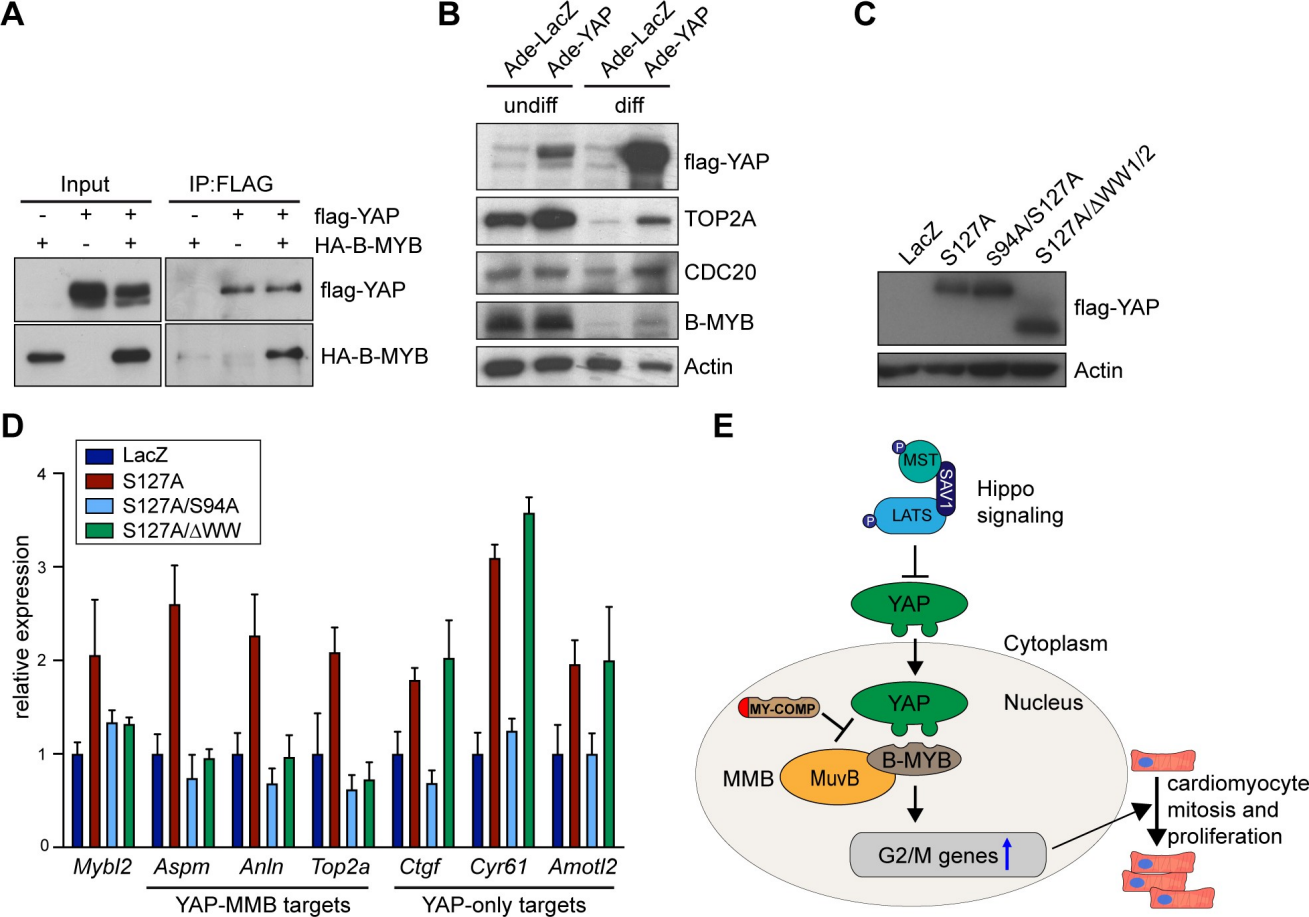
MMB target genes are downregulated in differentiated cells and re-activated by YAP. A) Co-immunoprecipitation of ectopically expressed flag-YAP and HA-B-MYB in C2C12 cells. IP, immunoprecipitation. Input: 3% of the amount used for IP was used. B) Undifferentiated C2C12 myoblasts (undiff) or differentiated C2C12 myotubes (diff) were infected with Ade-LacZ or Ade-YAP(S127A). After 2 days, the expression of the indicated proteins was analyzed by immunoblotting. Actin served as a loading control. C) and D) Differentiated C2C12 myotubes were infected with the indicated adenoviral expression constructs. C) The expression of the YAP constructs was analyzed by immunoblotting with a flag-antibody. Actin served as a loading control. D) mRNA expression of the indicated genes relative to *Actin* and *Hprt* was analyzed by RT-qPCR. Error bars show SD of technical triplicates from one representative experiment (n = 3). H) Model summarizing the results. See text for details. E), G) Error bars indicate SD. Student’s t-test. * = p<0.05, ** = p<0.01, *** = p<0.001, ns = not significant.

In another line of evidence, we investigated whether YAP is able to regulate MMB target genes in C2C12 cells. Co-immunoprecipitations confirmed that B-MYB and YAP interact in C2C12 myoblasts (Fig 9A). The MMB target genes CDC20 and TOP2A were expressed at high levels in asynchronously growing C2C12 cells and were downregulated during myogenic differentiation (Fig 9B). Expression of a constitutive active YAP(S127A) partially reverted the downregulation of these target genes in differentiated cells (Fig 9B). We next asked whether the re-induction of cell cycle genes in differentiated C2C12 cells is dependent on the YAP WW domains. C2C12 cells were differentiated into myotubes and then transduced with adenoviral constructs encoding for LacZ or for YAP mutants. The expression of YAP mutants was validated by immunoblotting (Fig 9C). Next, expression of cell cycle genes and YAP target gene was analyzed by RT-qPCR. Importantly, like the induction of proliferation of cardiomyocytes, the upregulation of MMB target genes in differentiated C2C12 cells by YAP was dependent on the WW and the TEAD-binding domains (Fig 9D). Strikingly activation of YAP target genes that are not regulated from distant enhancers but by binding of YAP to the proximal promoter, such as *Ctgf* and *Cyr61* and *Amotl2* (see Fig 2G and S2D Fig), did not require the WW domains, indicating that their induction is independent from binding to MMB (Fig 9D).

We next asked how the expression of B-MYB and MMB target genes is regulated during heart development. Hearts were collected at embryonic stage E16.5 and at postnatal days P1 and P10. mRNA was isolated and subjected to RT-qPCR. A panel of YAP-MMB target genes such as *Cenpf*, *Nusap1*, *Top2a* and *Birc5* were expressed at high levels in embryonic hearts when cardiomyocytes still divide but strongly declined in P1 and P10 hearts when cardiomyocytes differentiate and exit the cycle (S8A Fig). The downregulation of TOP2A, NUSAP1 and CENPF in adult hearts was also observed on protein level (S8B Fig). *Mybl2* also declined at P1 compared to E16.5 and further decreased at P10, a pattern that was also confirmed on protein level (S8A and S8B Fig). Thus, downregulation of MMB target genes correlates with the postnatal cell cycle exit of cardiomyocytes. Conversely, *Mybl2* and mitotic MMB target genes were expressed at higher levels in P1 *Sav1* knockout hearts when YAP is activated compared to wildtype hearts (S8C Fig). Together these data support the view that MMB contributes to the induction of cell cycle genes in response to Hippo-deficiency or YAP activation.

## Discussion

Recent studies have shown that the Hippo-YAP pathway plays essential roles during heart development and heart regeneration [4]. While the deletion of YAP in embryonic heart impairs cardiomyocyte division, the expression of constitutively active YAP promotes cardiomyocyte proliferation [5,9,33,34]. We now show that MMB is required for the ability of YAP to induce cell division in this tissue (Fig 9E). First, we used a mouse model in which cell cycle genes were induced in embryonic hearts by activation of YAP through the knockout of the Hippo pathway protein *Sav1*. These genes were not induced when the MuvB subunit *Lin9* was deleted together with *Sav1*. Proliferation of cardiomyocytes is also dependent on both MMB and YAP, since deletion of *Lin9* abolishes the enhanced proliferation induced by deletion of *Sav1*. This requirement is not limited to the embryonic heart but the same dependency on *Lin9* for cardiomyocyte proliferation was also observed in postnatal hearts using *Lin9* / *Sav1* double knockout mice expressing the Cre transgene from the alpha-MHC promoter. Further evidence for this dependency on LIN9 comes from experiments using cultured embryonal or postnatal cardiomyocytes with a conditional *Lin9* allele and a hormone inducible Cre recombinase. Adenoviral expression of activated YAP was only able to induce mitosis when *Lin9* was present but not after Cre-mediated deletion of *Lin9*. Although it is possible that YAP and MMB could independently induce a similar set of genes required for cell cycle regulation, our findings that B-MYB and YAP directly interact and that YAP induced cardiomyocyte proliferation depends on its WW domains supports the notion that the synergy is a result of the interaction between YAP and MMB. Furthermore, cardiomyocyte mitosis is inhibited by MY-COMP, a fragment of B-MYB that contains the YAP binding domain of B-MYB. When fused to a nuclear localization signal and expressed in cells, MY-COMP interferes with the association YAP to B-MYB.

Genome binding studies in embryonic hearts demonstrated that LIN9 binds to the promoters of cell cycle genes activated by YAP, indicating that these genes are direct targets of MMB. By ChIP-seq, YAP does not colocalize with LIN9 to the promoters of cell cycle genes but instead binds to enhancers, consistent with recent data from human cancer cell lines where YAP also predominantly binds to distant sites [24–26]. This suggests that YAP interacts with MMB-regulated promoters through chromatin looping, resulting in the activation of a subset of cell cycle genes, similar to what we have recently described for human MCF10A cells [14]. Given that YAP not only interacts with MMB but that the MMB subunit B-MYB is also a transcriptional target of YAP, one can speculate that YAP and B-MYB form a positive feedback loop enabling expression of downstream cell cycle genes.

Remarkably, although LIN9 was required for cardiomyocyte cell cycle induction due to activated YAP, LIN9 was completely dispensable for homeostasis of the adult murine heart. This absence of a phenotype in adult cardiac-specific *Lin9* knockout mice was surprising, because LIN9 is a key subunit of the DREAM complex that is involved in repression of cell cycle genes in non-dividing cells [15]. The observation that the genetic inactivation of *Lin9* in the postnatal heart is not associated with ectopic proliferation of cardiomyocytes suggests that loss of DREAM does not lead to sufficient de-repression of cell cycle genes to induce cardiomyocyte proliferation. It is therefore possible that DREAM does not permanently silence cell cycle genes, but keeps them in a poised state for re-activation by pro-proliferative signals, consistent with our finding that LIN9 remains bound to promoters in postnatal hearts. Also supporting this possibility is finding that cell cycle promoters, as opposed to promoters of genes that regulate cell-cell contacts and the cytoskeleton, are readily accessible in adult murine hearts before introduction of a YAP5SA transgene [6]. Thus, MuvB complexes may have a dual role in cardiomyocyte proliferation, contributing to the inactive, but primed state of cell cycle genes as well as to their YAP-mediated activation. It will be important to investigate whether epigenetic changes at MuvB bound promoters contribute to the loss of the regeneration capacity of adult hearts [35].

YAP not only plays a role in cardiac development but is also a potent oncogene in different cancers. Previous findings have implicated the YAP WW domain in oncogenic transformation [36]. Targeting WW domain mediated interactions by MY-COMP, or by more potent inhibitors based on MY-COMP, may prove a valid strategy to inhibit the oncogenic pro-proliferative functions of YAP. Such inhibitors could serve as selective therapeutic agents in cancers with high levels of YAP.

## Materials and methods

### Ethics statement

All animal studies were carried out in accordance with national and institutional guidelines and were approved by the regional state administration agency for Würzburg (approval number 55.2-2532-2-293). Euthanasia was by cervical dislocation of adults and decapitation of embryos.

### Mice

*Sav1*^*tm1*.*1Dupa*^/J mice were obtained from Jackson laboratories, in which LoxP sites flank exon 3 of the *Sav1* gene [23]. We have previously descripted *Lin9*^*f*l^ mice, in which LoxP sites flank exon 7 of the *Lin9* gene [37]. Cardiomyocyte-specific deletion of *Sav1* and *Lin9* was achieved by crossing mice to Nkx2.5-Cre mice [38]. Postnatal cardiomyocyte-specific deletion of *Sav1* and *Lin9* was achieved by crossing mice to α-MHC-Cre mice [27]. To obtain Lin9^fl/fl^CreER^T2^, *Lin9*^*fl*^ mice were crossed with a mouse line ubiquitously expressing CreER^T2^ transgene [39]. Mice were maintained in a C57/Bl6 background.

### Cell culture

HEK293A (Thermo Fisher Scientific) cells, HeLa (ATCC CCL-2, female) cells and C2C12 (ATCC CRL-1772) cells were maintained in DMEM supplemented with 10% FCS (Thermo Fisher Scientific) and 1% penicillin/streptomycin (Thermo Fisher Scientific). Differentiation of C2C12 cells was induced by culturing in DMEM medium with 2% horse serum (Sigma). Primary embryonic and postnatal cardiomyocytes were isolated by enzymatic digestion with the Pierce Primary cardiomyocyte isolation kit (Thermo Fisher Scientific). Cardiomyocytes were enriched by preplating on tissue-culture plastic to remove nonmyocytes. Cardiomyocytes were initially cultured for 48h on fibronectin-coated dishes with 5% horse serum (Sigma) and 1% penicillin/streptomycin (Thermo Fisher Scientific) and cardiomyocyte growth supplement at 37°C and 5% CO2 to prevent proliferation of nonmyocytes. To induce the deletion of *Lin9*, conditional cardiomyocytes were treated with 100 nM 4-hydroxytamoxifen (Sigma) for 4 days. Cardiomyocytes were transduced with adenovirus (multiplicity of infection, 25) in serum-free medium for 24 h and cultured for additional 48 h.

### Adenovirus

Adenoviral constructs were constructed using the ViralPower adenoviral expression system (Thermo Fischer Scientific). Entry vectors containing YAPS127A, YAPS94AS127A and YAPS127AΔ155–263 were generated by PCR from p2xFLAG-CMV-YAPS127A [40]. Entry vectors containing GFP, HA-NLS-B-MYB (2–241)-T2A-GFP and HA-NLS-B-MYB (2–241, ΔPPXY)-T2A-GFP were generated by PCR from pCDNA4/TO-HA-NLS-SV40-B-MYB (this work) and pSpCas9n(BB)-2A-GFP (PX461) (Addgene #48140). All entry vectors were subcloned into pENTR3C (Invitrogen) and then recombined into pAd/CMV/V5-DEST using LR clonase II (Thermo Fischer Scientific). The production of YAP mutant and LacZ adenoviruses was performed in HEK293A cells according to the manufacturer’s instructions. Adenoviruses were titered using the Adeno-X Rapid Titer Kit (TaKaRa).

### RT-qPCR

Total RNA was isolated using peqGOLD TriFast (Peqlab) according to the manufacturer’s instructions. RNA was transcribed using 100 units RevertAid reverse transcriptase (Thermo Fisher Scientific). Quantitative real–time PCR reagents were from Thermo Fisher Scientific and real-time PCR was performed using the Mx3000 (Stratagene) detection system. Primer sequences are listed in S1 Table. Expression differences were calculated as described before [20].

### PLA

PLA was performed using the Duolink In Situ Kit (Sigma) according to the manufacturer’s instructions. The following antibodies were used: LIN9 (Bethyl, A300-BL2981; 1:150), B-MYB phospho-T487 (ab76009; 1:100), YAP (Santa Cruz Biotechnology, sc-101199; 1:200). For secondary staining, samples were incubated for 45 minutes with the following antibodies: cTnT (DSHB; 1:50) or α-HA (Sigma, 1:200). After washing several times with buffer A, samples were incubated with for 20 minutes with secondary antibody: α-IgG2a conjugated to Alexa 488 (Thermo Fisher Scientific) and Hoechst 33258 (Sigma). Pictures were taken with an inverted Leica DMI 6000B microscope equipped with a Prior Lumen 200 fluorescence light source and a Leica DFC350 FX digital camera.

### Plasmids

Expression vectors for truncated human FLAG-YAP constructs (aa 2–499, 2–287, 2–263, 2–154, 155–504, 264–504, 288–504 and Δ155–263) were constructed from pCMV-2xFLAG-YAP [41] by PCR. Expression vector for human GST-YAP constructs (aa 2–263, 155–263, 168–204, 231–263) were constructed by PCR in the pGEX-4-T2 vector. Truncated human HA-B-MYB constructs (aa 2–410, 2–331, 80–331, 80–410, 2–241, 2–79, 242–410) were generated from pCDNA4/TO-HA-B-MYB. Expression vector for truncated human 6xHis-B-MYB-2-241 and 2–241Δ198–201 (ΔPPXY) were constructed by PCR using appropriate oligonucleotides and were subcloned into pRSETA. Expression vectors containing HA-NLS-SV40-B-MYB-2-241, 2–241 N174A(ΔDNA), 2–241 Δ198-201(ΔPPXY) were obtained by PCR and were subcloned into pCDNA4/TO vector.

### Immunoblotting and immunoprecipitation

Whole protein extracts were obtained by lysing cells in TNN buffer (50mM Tris (pH 7.5), 120mM NaCl, 5mM EDTA, 0.5% NP40, 10mM Na4P_2_O_7_, 2mM Na_3_VO_4_, 100mM NaF, 1mM PMSF, 1mM DTT, 10mM β-glycerophosphate, protease inhibitor cocktail (Sigma)). Protein lysates or purified protein were separated by SDS-PAGE, transferred to a PVDF-membrane and detected by immunoblotting with the first and secondary antibodies: β-actin (Santa Cruz Biotechnology, sc-47778) 1:5000, B-MYB (clone LX015.1, [42] 1:5, anti-HA.11 (Covance, MMA-101P) 1:1000, anti-FLAG M2 (Sigma, F3165) 1:5000, anti-His (Sigma, H1029) 1:2000, Vinculin (Sigma, V9131) 1:10000, TOP2A (Santa Cruz Biotechnology, sc-365916) 1:1000, CDC20 (Santa Cruz Biotechnology, sc-5296) 1:500, YAP (Santa Cruz Biotechnology, sc-10199) 1:1000, p-YAP(S127A) (Cell Signaling Technology, 4911) 1:1000, LIN9 (Bethyl, A300-BL2981), NUSAP1 (Geert Carmeliet) 1:1000, CENPF (Abcam, ab-5) 1:1000, anti-mouse-HRP (GE healthcare, NXA931) 1:5000 and HRP Protein A (BD Biosciences, 610438) 1:5000. For immunoprecipitation of FLAG-tagged proteins, protein G dynabeads (Thermo Fisher Scientific) were first coupled with 1 μg FLAG-antibody (Sigma, F3165) and then immunoprecipitated with 1mg whole cell lysate. After five times of washing with TNN, proteins were separated by SDS-PAGE and detected by immunoblotting using the desired antibodies.

### Immunostaining

For immunostaining cardiomyocytes were seeded onto fibronectin coated coverslips. HeLa cells were seeded onto coverslips without fibronectin. Cells were fixed with 3% paraformaldehyde and 2% sucrose in PBS for 10 minutes at room temperature. Cells were permeabilized using 0.2% Triton X-100 (Sigma) in PBS for 5 minutes and blocked with 3% BSA in PBS-T (0.1% Triton X-100 in PBS) for 30 minutes. Primary antibodies were diluted in 3% BSA in PBS-T and incubated with the cells for 1 hour at room temperature. The following antibodies were used: TroponinT (CT3) (developmental studies hybridoma bank) 1:50, phospho-Histone H3 (Ser10) (Santa Cruz Biotechnology, sc-8656) 1:100, Ki-67 (SP6) (Thermo Scientific, RM-9106) 1:200, α-tubulin (B-5-1-2) (Santa Cruz Biotechnology, sc-23948) 1:150 and anti-HA (Sigma, H6908) 1:200. After three washing steps with PBS-T, secondary antibodies conjugated to Alexa 488 and 594 (Thermo Fisher Scientific) and Hoechst 33258 (Sigma) were diluted 1:500 in 3% BSA in PBS-T and incubated with the coverslips for 30 minutes at room temperature. Finally, slides were washed three times with PBS-T and mounted with Immu-Mount (Thermo Fisher Scientific). Pictures were taken with an inverted Leica DMI 6000B microscope equipped with a Prior Lumen 200 fluorescence light source and a Leica DFC350 FX digital camera.

### Histology, H&E staining and immunohistochemistry

Freshly dissected embryos and postnatal hearts were fixed with bouin’s fixative (picric acid (saturated; AppliChem), 10% (v/v) formaldehyde, 5% (v/v) (AppliChem) glacial acetic acid (Roth) embedded in paraffin and sectioned. Sections were deparaffinized, rehydrated and stained with hematoxylin and eosin or processed for immunostaining.

Following deparaffinization, antigen retrieval was performed by boiling samples in 10mM sodium citrate buffer (pH6.0) for 10 minutes in a microwave oven. After 30 minutes cool down, samples were blocked with 3% BSA in PBS-T (0.1% Tween-20 (AppliChem)) and incubated with the primary antibodies diluted in PBS-T over night at 4°C. The following antibodies were used: TroponinT (CT3) (developmental studies hybridoma bank) 1:50, phospho-Histone H3 (Ser10) (Santa Cruz Biotechnology, sc-8656) 1:100, Ki-67 (SP6) (Thermo Scientific, RM-9106) 1:200. After three washing steps with PBS-T, secondary antibodies conjugated to Alexa 488 and 594 (Thermo Fisher Scientific) and Hoechst 33258 (Sigma) were diluted 1:200 in 3% BSA in PBS-T and incubated with the coverslips for 2 hours at room temperature. Finally, slides were washed three times with PBS-T and mounted with Immu-Mount (Thermo Fisher Scientific). Pictures were taken with an inverted Leica DMI 6000B microscope equipped with a Prior Lumen 200 fluorescence light source and a Leica DFC350 FX digital camera.

For morphometric measurements, we measured transverse sections at the level of atrioventricular valves. Wall thickness was calculated as the ventricular compact myocardial thickness divided by its outer circumference. Myocardial area was quantified in each section using Image J as previously described [43].

### siRNA transfection of embryonal cardiomyocytes

Double-stranded RNA was purchased from Eurofins. siRNAs were transfected in a final concentration of 30 nM using RNAiMAX (Thermo Fisher Scientific) according to the manufacturer’s protocol. siRNA sequences are listed in S1 Table [44, 45].

### Flow cytometry

Cardiomyocytes were isolated and enriched with the Pierce Primary cardiomyocyte isolation kit (Thermo Fisher Scientific) according to the manufacturer’s instructions. Isolated and enriched cardiomyocytes were fixed with 4% PFA in PBS for 5 minutes. Detection was performed using a Beckman Coulter FC 500 cytomics and data were analyzed in CXP analysis 2.2 software. Gating and compensation were based on fluorophore-negative controls. 10.000 cells were analyzed per genotype.

### RNA-Seq

For whole transcriptome analysis, total RNA was isolated in triplicates from ventricular heart tissue with the desired genotype. DNA libraries were generated using 1μg RNA with the magnetic mRNA isolation module and NEBNext Ultra II RNA Library Prep Kit for Illumina (New England Biolabs). DNA library was amplified by 7 PCR cycles and quality was analyzed using the fragment analyzer (Advanced Analytical). Libraries were sequenced on the NextSeq 500 platform (Illumina).

### ChIP-Seq

Chromatin was isolated from ventricular heart tissue of E16.5 or P10 hearts. In brief, minced tissue was fixed with 1% formaldehyde for 20 minutes at RT. The reaction was stopped by adding 125mM glycine for additional 5 minutes. Tissue was incubated in lysis buffer (50mM Tris-HCl pH8, 10mM EDTA, 1% SDS) for 1 hour at 4°C. Samples were sonicated for 1 minute at 25% amplitude (10s ON / 30s OFF) at 4°C, insoluble material was removed by centrifugation and chromatin was fragmented to an approximate size of 150 to 300bp by additional sonicating for 10 minutes at 25% amplitude (10 seconds On/ 30 seconds OFF) at 4°C using a Branson sonifier. Afterwards chromatin was diluted ten times in ChIP dilution buffer (50mM Tris-HCl pH8, 0.167M NaCl, 1.1% Triton X-100, 0.11% sodium deoxycholate). For immunoprecipitation, 9μg of the antibody was coupled to protein G dynabeads (Thermo Fisher Scientific) for 6 hours at 4°C and then incubated with fragmented chromatin over night at 4°C. Beads were washed in total twelve times with wash buffer I (50mM Tris-HCl pH8, 0.15M NaCl, 1mM EDTA, 0.1% SDS, 1% Triton X-100, 0.1% sodium deoxycholate), wash buffer II (50mM Tris-HCl pH8, 0.5M NaCl, 1mM EDTA, 0.1% SDS, 1% Triton X-100, 0.1% sodium deoxycholate), wash buffer III (50mM Tris-HCl pH8, 0.5M LiCl_2_, 1mM EDTA, 1% Nonidet P-40, 0.7% sodium deoxycholate) and wash buffer IV (10mM Tris-HCl pH8, 1mM EDTA). 1mM PMSF and protease inhibitor cocktail were added freshly to all buffers. After washing chromatin was eluted in (10mM Tris-HCl pH8, 0.3M NaCl, 5mM EDTA, 0.5% SDS, 10μg/ml RNAseA) and crosslink was reversed at 65°C over night. Proteins were digested by adding 200μg/ml proteinase K at 55°C for 2 hours. DNA was purified using the QIAquick PCR Purification Kit (QIAGEN) and eluted in 50 μl EB buffer. Purified ChIP-DNA was quantified using the Quant-iT PicoGreen dsDNA Assay Kit (Thermo Fisher Scientific). DNA libraries were generated using 10ng purified ChIP-DNA and the NEBNext Ultra IIDNALibrary Prep Kit for Illumina (New England Biolabs) according to the manufacturer’s instructions. DNA library was amplified by 12–15 PCR cycles and quality was analyzed using the fragment analyzer (Advanced Analytical). Libraries were sequenced on the NextSeq 500 platform (Illumina). The following antibodies were used for ChIP-seq: LIN9 (Bethyl, A300-BL2981), YAP (NB110-58358) and IgG from rabbit serum (Sigma, I5006).

### GST-pulldown

Recombinant GST, GST-TEAD-WW1/2 (TEAD-binding domain and WW domain 1 and 2 of YAP fused to GST), GST-WW1/2-, GST-WW1, GST-WW2 or His-B-MYB(2–241) (amino acids 2–241 fused to 6 x his) were expressed in BL21 cells and purified on glutathione-linked sepharose beads (GE healthcare) or Ni-NTA-Agarose (GE healthcare, His-B-MYB). Lysates of HeLa cells expressing HA-tagged B-MYB constructs were incubated with 5 mg immobilized GST or GST-WW1/2-YAP for 3 hours at 4°C. Beads were washed six times with TNN buffer, resuspended in SDS protein sample buffer, boiled for 5 min, separated on a 10% SDS-PAGE gel, blotted and analyzed via immunoblotting.

### Peptide microarrays

μSPOT peptide arrays [46] (CelluSpots, Intavis AG, Cologne, Germany) were synthesized using a MultiPep RSi robot (Intavis AG) on in-house produced, acid labile, amino functionalized, cellulose membrane discs containing 9-fluorenylmethyloxycarbonyl-β-alanine (Fmoc-β-Ala) linkers (average loading: 131 nmol/disc– 4 mm diameter). Synthesis was initiated by Fmoc deprotection using 20% piperidine in dimethylformamide (DMF, 2×2 μL per disc for 10 min each) followed by washing with DMF (6×400 μL per disc) and ethanol (EtOH, 2×400 and 2×600 μL per disc). Peptide chain elongation was achieved using 1.2 μL of coupling solution, consisting of preactivated amino acids (aas, 0.5 M) with ethyl 2-cyano-2-(hydroxyimino)acetate (oxyma, 1 M) and *N*,*N′*-diisopropylcarbodiimide (DIC, 1 M) in DMF (2:1:1, aa:oxyma:DIC). Couplings were carried out for 2×30 min, and subsequently, the membrane was capped with capping mixture (4% acetic anhydride in DMF, 2×2 μL/disc for 5 min each), followed by 7× washes with 300 μL DMF/disc. Synthesis was finalized by deprotection with 20% piperidine in DMF (2×4 μL/disc for 10 min each), followed by washing with DMF (7×400 μL/disc) and EtOH (2×400 μL/disc, 2×600 μL/disc, 3×400 μL/disc). Dried discs were transferred to 96 deep-well blocks and treated with sidechain deprotection solution, consisting of 90% trifluoracetic acid (TFA), 2% dichloromethane (DCM), 5% H_2_O and 3% triisopropylsilane (TIPS) (150 μL/well) for 1.5 h at room temperature (rt). Afterwards, the deprotection solution was removed, and the discs were solubilized overnight at rt using a solvation mixture containing 88.5% TFA, 4% trifluoromethanesulfonic acid (TFMSA), 5% H_2_O and 2.5% TIPS (250 μL/well). The resulting peptide-cellulose conjugates (PCCs) were precipitated with ice-cold ether (0.7 mL/well) and spun down at 500×g for 10 min at 4°C, followed by two additional washes of the formed pellet with ice-cold ether. The resulting pellets were dissolved in DMSO (250 μL/well) to give final stocks. PCC solutions were mixed 2:1 with saline-sodium citrate (SSC) buffer (150 mM NaCl, 15 mM trisodium citrate, pH 7.0) and transferred to a 384-well plate. For transfer of the PCC solutions to white coated CelluSpot blank slides (76×26 mm, Intavis AG), a SlideSpotter (Intavis AG) was used. After completion of the printing procedure, slides were left to dry overnight.

μSPOT slides were blocked by incubation with 2% (w/v) BSA in PBS for 60 min. Afterwards, slides were incubated with YAP-WW1/2 or GST protein in 0.1% BSA in PBS for 30 min. Slides were washed 6 times with PBS for 5 min and then incubated with 1:10,000 diluted primary antibody (Anti-GST HRP conjugated (RPN1236V; Sigma Aldrich) in 0.1% BSA in PBS for 30 min, after which the slides were washed 6 times with PBS for 5 min. Peptide binding was detected through chemiluminescent detection with SuperSignal West Femto Maximum Sensitive Substrate (Thermo Scientific) using a c400 imaging system (Azure). Binding intensities were evaluated using FIJI including the Microarray Profile addon (OptiNav). The error range and standard deviation were defined by comparing the intensities of each peptide duplicate on the respective array.

### Bioinformatic analysis

After sequencing, bases were called using Illuminas GenerateFASTQ v1.1.0.64 software and sequencing quality was controlled with the FastQC script. For RNA-seq, reads were mapped with TopHat v2.1.0 [47] and BOWTIE2 [48] with default parameters to the murine genome (mm10). Samples were normalized to the sample with the smallest number of mappable reads and a read count matrix for each Ensembl gene (GRCm38.p6) was generated with the *summarizeOverlaps* function in the R package {GenomicFeatures}. Before differential gene expression analysis, non- or weakly expressed genes were removed using the threshold: mean read count per gene over all samples >1. Differentially expressed genes were called with *EdgeR* and p-values were adjusted for multiple-testing with the Benjamini-Höchberg procedure (FDR: false discovery rate). Gene set enrichment analyses were performed with signal2noise metric, 1000 permutations and a combined gene set database comprising Hallmark and C2 gene sets.

For ChIP-seq, sequenced reads were mapped to the *Mus musculus* genome mm10 with BOWTIE v1.2 [48] with default parameters and subsequently normalized to the sample with the smallest number of sequenced reads. Peaks were called with MACS14 [49] with maximal 3 duplicates, a p-value cut-off of 1e-5 and the input sample as control. Resulting peaks were annotated to the next transcriptional start of Ensembl genes with the *closestBed* function from the bedtools suite v2.26.0 [50]. Overlapping peaks were identified with *bedtools intersect* and a minimal overlap of 1bp. LIN9 occupancy was calculated in a window of +/-1kb around TSSs with *bedtools coverage* function. Density matrices were generated with deeptools v2.3.5. [51] *computeMatrix* function at a resolution of 1bp and subsequently used for plotting heat maps with *plotHeatmap*. Mapped ChIP-seq data for histone marks were taken from the ENCODE portal [52] (https://www.encodeproject.org/) with the following identifiers: ENCFF056JGV, ENCFF295HNV, ENCFF642EEK, ENCFF687BWU. Promoters, enhancers and super-enhancers were defined as described previously [14].

In box plots, the central line reflects the median, the borders of the boxes indicate the first and third quartile and whiskers extend to 1.5 of the interquartile range. Outliers are shown as dots. P-values were calculated with a two-tailed Wilcoxon rank sum test (unpaired samples) or Wilcoxons signed-rank test (paired samples). ChIP- and RNA-sequencing datasets are available at the NCBI’s Gene Expression Omnibus [53] under the accession number GEO: GSE137132.

## Supporting information

S1 Fig Proliferation of embryonic cardiomyocytes following Hippo inactivation depends on LIN9. A) Expression of YAP and levels of YAP phosphorylated on S127 (p-YAP) in the hearts of *Nkx2*.*5-Cre; Sav1*^*+/+*^ and *Nkx2*.*5-Cre; Sav1*^*fl/fl*^ mice was determined by immunoblotting. Actin served as control. B) C): Lower magnification overview pictures of heart sections of E13.5 mice stained for Ki67 (B) or phospho-H3 (C). Scale bar: 200 μm. See Fig 1A and 1C.
(TIF)

S2 Fig Cell cycle genes upregulated in *Sav1* knockout hearts are direct targets of LIN9. A) Representative gene sets from the analysis in Fig 5A. p-values were calculated using a permutation test with 1000 permutations. „Signal2Noise”was used as a metric to rank genes. ES: enrichment score. B) Heatmap depicting the mRNA expression of LIN9 regulated cell cycle genes in hearts of *Nkx2*.*5-Cre* mice with the indicated genotypes as determined by RNA-seq. C) Heat map documenting binding of LIN9 and YAP at LIN9 peaks in promoters or at YAP peaks in enhancers and superenhancers in E16.5 heart ventricles. Read density is plotted in a window of +/-2kb around the peak at a resolution of 2bp. Data for histone modifications are taken from ENCODE. D) Genome browser tracks illustrating the binding of LIN9 to the Mybl2, Anln and Top2a promoter and binding of YAP to the Cyr61 and Ctgf promoter and to an intergenic enhancer on chromosome 1. ChIP-seq data for histone modifications are from ENCODE (GSE31039). E) GSEA comparing expression differences in *Nkx2*.*5-Cre; Lin9*^*fl/fl*^ (LIN9 KO) and *Nkx2*.*5-Cre; Lin9*^*fl/+*^ (LIN9 wt) heart ventricles from E13.5 mice in two biological replicates (each done in triplicate). The C2 MSigDB was spiked with the Hallmark gene sets and a set of LIN9 direct targets genes from [14]. Gene sets related to respiration/TCA cycle (“oxphos“) and hematopoietic cells are highlighted in blue and orange, respectively. NES: normalized enrichment score. F) Representative gene sets from the analysis in C. p-values were calculated using a permutation test with 1000 permutations. „Signal2Noise”was used as a metric to rank genes. ES: enrichment score.
(TIF)

S3 Fig LIN9 is required for cardiomyocyte proliferation in Hippo-deficient, postnatal hearts. A) Embryonic lethality of *Nkx2*.*5*^*Cre*^*; Lin9*^*fl/fl*^ mice. Breeding scheme and resulting genotypes. Result of the genotyping of live embryos at the indicated developmental time points. B) H&E-stained sections of embryonic E13.5 hearts of mice with the indicated genotypes. RA: right atrium, RV: right ventricle, LA: left atrium, LV: left ventricle, IVS: interventricular septum. Scale bar: 500μm C) Viability of *α-MHC-Cre; Lin9*^*fl/fl*^ mice. Breeding scheme and resulting genotypes. Number of mice with the indicated genotypes at P10 and P21-25. D) Example FACS data of mTomato and mEGFP positive cardiomyocytes derived from hearts of E13.5 and P10 hearts with the indicated genotypes. See Fig 3G. E) The expression of *Lin9* relative to *Actin and Hprt* was investigated in E16.5, P1 and P10 hearts by RT-qPCR. n = 3 independent replicates. F) The expression of LIN9 in lysates prepared from hearts at the different developmental stages was investigated by immunoblotting. β-actin served as a loading control. G) Heat map documenting binding of LIN9 at LIN9 peaks in promoters called in E16.5 or P10 cardiomyocytes or overlapping peaks. Read density is plotted in a window of +/-2kb around the peak at a resolution of 2bp. Data for histone modifications are taken from ENCODE (GSE31039). H) Venn diagram depicting the common LIN9 peaks in E16.5 and P10 hearts. The number in brackets refers to the peaks located in promoters. I) Plot illustrating the genomic localization of LIN9 in postnatal (P10) heart ventricles as determined by ChIP-seq. J) Histogram showing the absolute distance between overlapping LIN9 peaks called in E16.5 and P10 heart ventricles located in promoters (n = 1,458) at a resolution of 20 bp.
(TIF)

S4 Fig Cardiomyocyte proliferation by activated YAP requires MMB. A) -B) Embryonal (E14.5) *Lin9*^*fl/fl*^; *CreER*^*T2*^ cardiomyocytes (A) or postnatal (P1) cardiomyocytes (B) were transduced with Ade-LacZ or with Ade-YAP[S127A] and treated with or without 4-OHT. The fraction of pH3-positive cardiomyocytes was quantified by staining for pH3 (red). Scale bar: 25 μm. Example microphotograph of the experiments shown in Fig 4C and 4D.
(TIF)

S5 Fig The WW domains of YAP mediate the interaction with B-MYB and are required to induce cardiomyocyte proliferation. A) Densiometric quantification of binding data shown in Fig 6B using ImageJ. Binding is relative to HA-B-MYB control cells. n = 3 biological replicates. B) Scheme of the GST fusion constructs used in pulldown experiments in Fig 6D and S5C Fig C) Pulldown experiments of the indicated GST fusion proteins with HA-B-MYB. Bound B-MYB was detected by immunoblotting with an HA-antibody. Input: 3% of the lysate used for the pulldown was loaded onto the gel. Actin served as a control. Ponceau staining was used to detect the recombinant GST-proteins.
(TIF)

S6 Fig Disrupting the association between B-MYB and YAP by MY-COMP. A) μSPOT based mapping of YAP WW1/2 interactions. An overlapping peptide library to display the whole B-MYB protein was probed with only Anti-GST-HRP, with purified recombinant GST and Anti-GST-HRP (control) or with purified recombinant GST-WW1/2 and Anti-GST-HRP. Binding was detected by chemiluminescene. Most prominent binding of YAP is observed at position E1. The respective B-MYB derived peptide contains a WW-binding PPXY motif. B) Zoomed view of marked region in A showing spots corresponding to the most prominent binding sequences within B-MYB with relative binding intensities and standard deviation (SD). C) Co-immunoprecipitation experiments of the indicated B-MYB constructs with flag-YAP. Lysates of HeLa cells expressing flag-YAP and HA-B-MYB (wt and ΔPPXY) were immunoprecipitated with flag-antiserum and immunoblotted with an anti-HA-antibody and anti-flag antibody. 3% percent of total lysate was immunoblotted (Input). D) Densiometric quantification of the binding of HA-MYB and HA-B-MYBΔPPXY to flag-YAP using Image J. Binding is relative to HA-B-MYB control cells. E) Densiometric quantification of the binding of HA-B-MYB to flag-YAP in presence and absence of MY-COMP using Image J. Binding is relative to FLAG-YAP control cells. N = 3 biological replicates. F) Proximity ligation assay (PLA) of endogenous YAP and B-MYB upon transfection of MY-COMP. Cell expressing MY-COMP were identified by HA staining (green). Example microphotographs of the experiments shown in Fig 7F. D), E): Error bars indicate SEMs. Student’s t-test. * = p<0.05, ** = p<0.01.
(TIF)

S7 Fig The YAP-B-MYB interaction is required for cardiomyocyte proliferation. A) and B) Cardiomyocytes were transduced with Ade-LacZ, Ade-YAP[S127A], and Ade-YAP[S127A/S94A] or with Ade-YAP[S127A/ΔWW]. The fraction of pH3-positive positive (A) and Ki67 (B) cardiomyocytes was determined by immunostaining. Example microphotographs of the experiment shown in Fig 6E and 6F. Scale bar: 25 μm. C) Embryonal cardiomyocytes were infected with adenoviruses expressing GFP, MY-COMP, with MY-COMP,ΔPPXY or with MY-COMP,ΔDNA each coupled to GFP through a T2A self-cleaving peptide. Mitotic cells were quantified by staining for phospho-H3 (red). Scale bar: 25 μm.
(TIF)

S8 Fig B-MYB and MMB target gene expression is downregulated during heart development. A) The expression of the indicated MMB target genes relative to *Hprt and Actin* was investigated in E16.5, P1 and P10 hearts. n = 3 independent replicates. B) The expression of the indicated proteins in lysates prepared from hearts at the different developmental stages was investigated by immunoblotting. β-Actin served as a control. C) Expression of the indicated genes in *Nkx2*.*5-Cre; Sav1*^*+/+*^ and *Nkx2*.*5-Cre; Sav1*^*fl/fl*^ in hearts of P1 mice was investigated. n = 7 independent *Nkx2*.*5-Cre; Sav1*^*+/+*^ and *Nkx2*.*5-Cre; Sav1*^*fl/fl*^ animals.
(TIF)

S9 Fig Original blot and gel images. Part 1.
(TIF)

S10 Fig Original blot and gel images. Part 2.
(TIF)

S11 Fig Original blot and gel images. Part 3.
(TIF)

S1 Table List of RT-qPCR primers for murine genes and of siRNA sequences. (XLSX)
