# Supplementary figures and images for: Interaction of YAP with the Myb-MuvB (MMB) complex defines a transcriptional program to promote the proliferation of cardiomyocytes
Source: PLoS Genet. 2020 May 29;16(5):e1008818. doi: 10.1371/journal.pgen.1008818 (PMC7286521; doi:10.1371/journal.pgen.1008818)

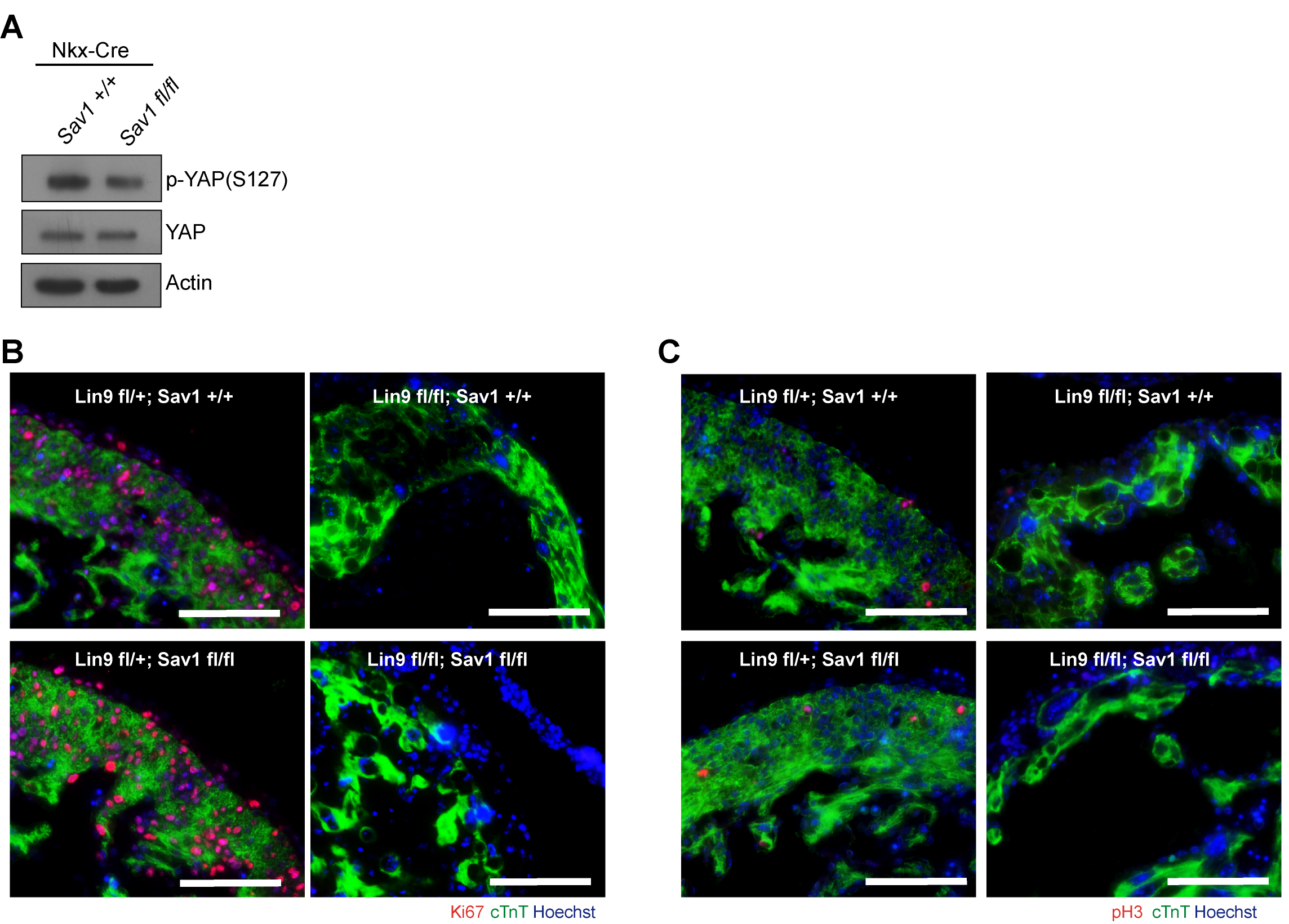

Supplement: S1 Fig — A) Expression of YAP and levels of YAP phosphorylated on S127 (p-YAP) in the hearts of Nkx2.5-Cre; Sav1+/+ and Nkx2.5-Cre; Sav1fl/fl mice was determined by immunoblotting. Actin served as control. B) C): Lower magnification overview pictures of heart sections of E13.5 mice stained for Ki67 (B) or phospho-H3 (C). Scale bar: 200 μm. See Fig 1A and 1C. (TIF) [file pgen.1008818.s001.tif]

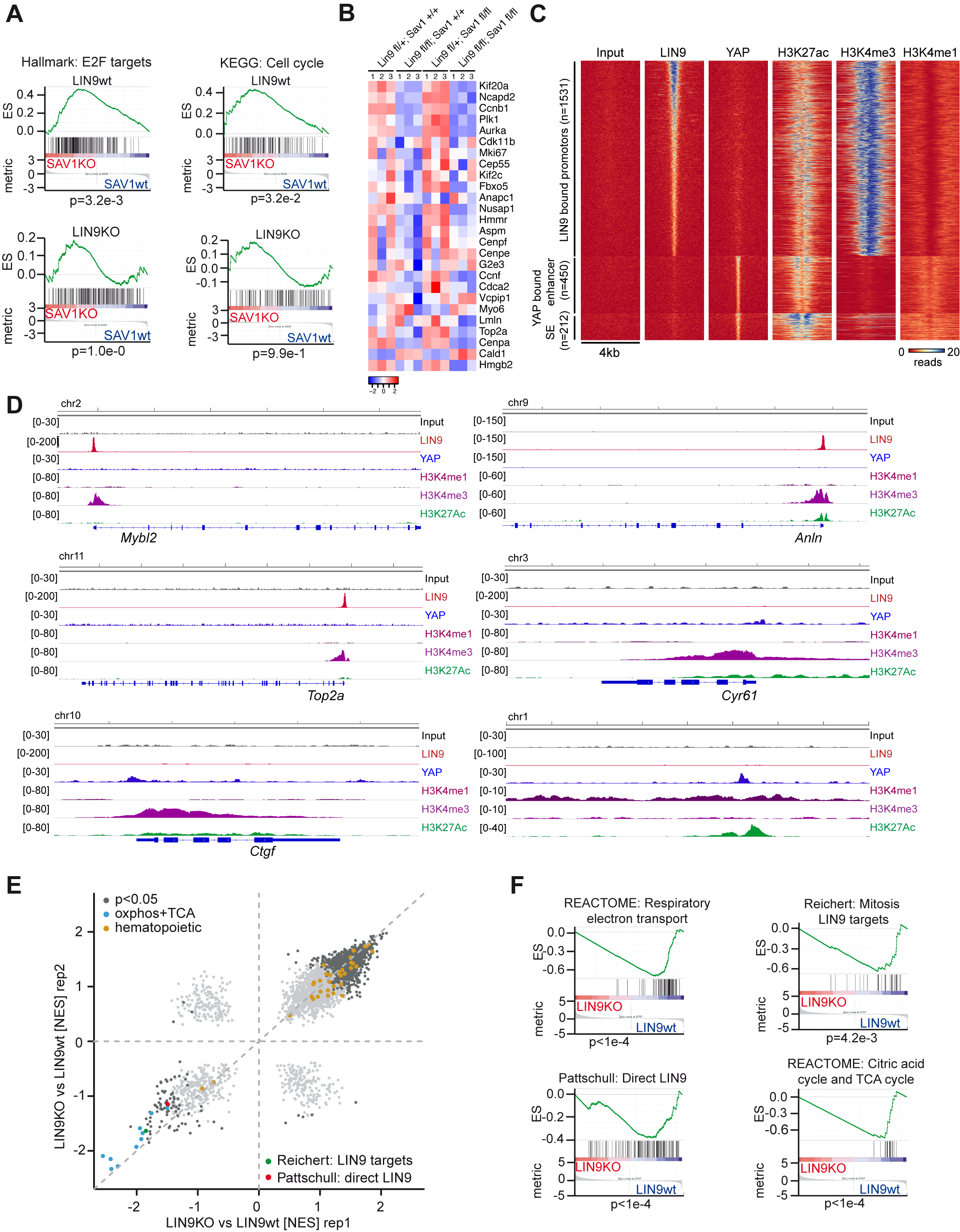

Supplement: S2 Fig — A) Representative gene sets from the analysis in Fig 5A. p-values were calculated using a permutation test with 1000 permutations. „Signal2Noise”was used as a metric to rank genes. ES: enrichment score. B) Heatmap depicting the mRNA expression of LIN9 regulated cell cycle genes in hearts of Nkx2.5-Cre mice with the indicated genotypes as determined by RNA-seq. C) Heat map documenting binding of LIN9 and YAP at LIN9 peaks in promoters or at YAP peaks in enhancers and superenhancers in E16.5 heart ventricles. Read density is plotted in a window of +/-2kb around the peak at a resolution of 2bp. Data for histone modifications are taken from ENCODE. D) Genome browser tracks illustrating the binding of LIN9 to the Mybl2, Anln and Top2a promoter and binding of YAP to the Cyr61 and Ctgf promoter and to an intergenic enhancer on chromosome 1. ChIP-seq data for histone modifications are from ENCODE (GSE31039). E) GSEA comparing expression differences in Nkx2.5-Cre; Lin9fl/fl (LIN9 KO) and Nkx2.5-Cre; Lin9fl/+ (LIN9 wt) heart ventricles from E13.5 mice in two biological replicates (each done in triplicate). The C2 MSigDB was spiked with the Hallmark gene sets and a set of LIN9 direct targets genes from [14]. Gene sets related to respiration/TCA cycle (“oxphos“) and hematopoietic cells are highlighted in blue and orange, respectively. NES: normalized enrichment score. F) Representative gene sets from the analysis in C. p-values were calculated using a permutation test with 1000 permutations. „Signal2Noise”was used as a metric to rank genes. ES: enrichment score. (TIF) [file pgen.1008818.s002.tif]

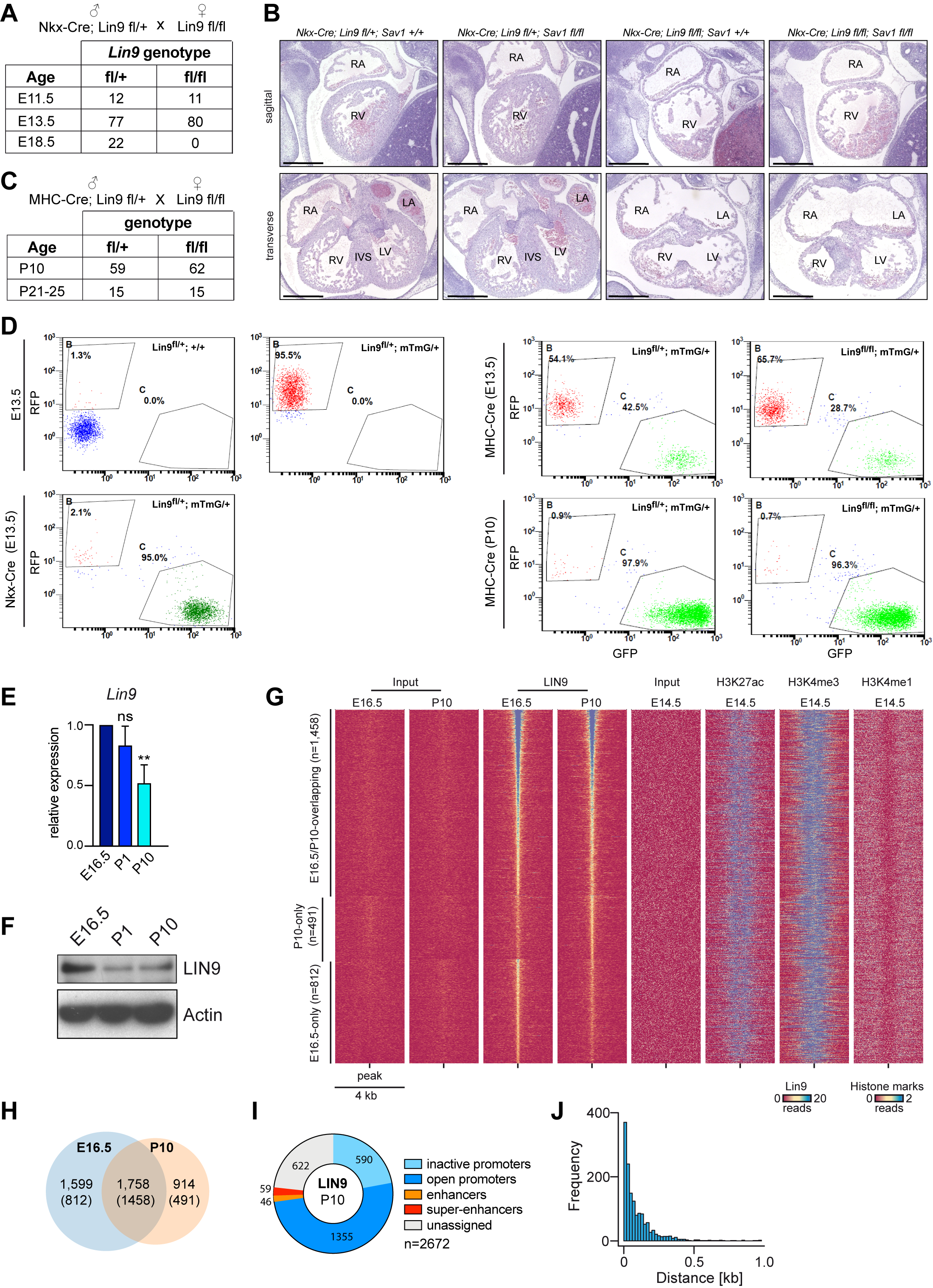

Supplement: S3 Fig — A) Embryonic lethality of Nkx2.5Cre; Lin9fl/fl mice. Breeding scheme and resulting genotypes. Result of the genotyping of live embryos at the indicated developmental time points. B) H&E-stained sections of embryonic E13.5 hearts of mice with the indicated genotypes. RA: right atrium, RV: right ventricle, LA: left atrium, LV: left ventricle, IVS: interventricular septum. Scale bar: 500μm C) Viability of α-MHC-Cre; Lin9fl/fl mice. Breeding scheme and resulting genotypes. Number of mice with the indicated genotypes at P10 and P21-25. D) Example FACS data of mTomato and mEGFP positive cardiomyocytes derived from hearts of E13.5 and P10 hearts with the indicated genotypes. See Fig 3G. E) The expression of Lin9 relative to Actin and Hprt was investigated in E16.5, P1 and P10 hearts by RT-qPCR. n = 3 independent replicates. F) The expression of LIN9 in lysates prepared from hearts at the different developmental stages was investigated by immunoblotting. β-actin served as a loading control. G) Heat map documenting binding of LIN9 at LIN9 peaks in promoters called in E16.5 or P10 cardiomyocytes or overlapping peaks. Read density is plotted in a window of +/-2kb around the peak at a resolution of 2bp. Data for histone modifications are taken from ENCODE (GSE31039). H) Venn diagram depicting the common LIN9 peaks in E16.5 and P10 hearts. The number in brackets refers to the peaks located in promoters. I) Plot illustrating the genomic localization of LIN9 in postnatal (P10) heart ventricles as determined by ChIP-seq. J) Histogram showing the absolute distance between overlapping LIN9 peaks called in E16.5 and P10 heart ventricles located in promoters (n = 1,458) at a resolution of 20 bp. (TIF) [file pgen.1008818.s003.tif]

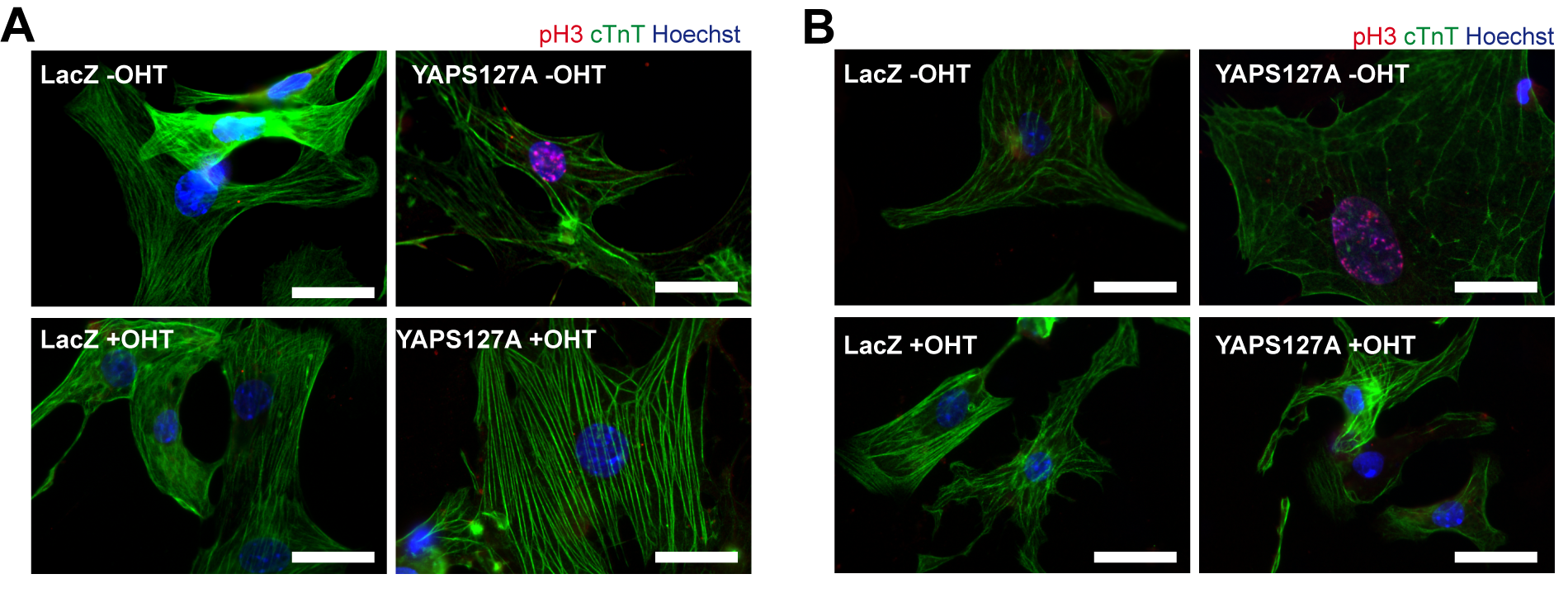

Supplement: S4 Fig — A) -B) Embryonal (E14.5) Lin9fl/fl; CreERT2 cardiomyocytes (A) or postnatal (P1) cardiomyocytes (B) were transduced with Ade-LacZ or with Ade-YAP[S127A] and treated with or without 4-OHT. The fraction of pH3-positive cardiomyocytes was quantified by staining for pH3 (red). Scale bar: 25 μm. Example microphotograph of the experiments shown in Fig 4C and 4D. (TIF) [file pgen.1008818.s004.tif]

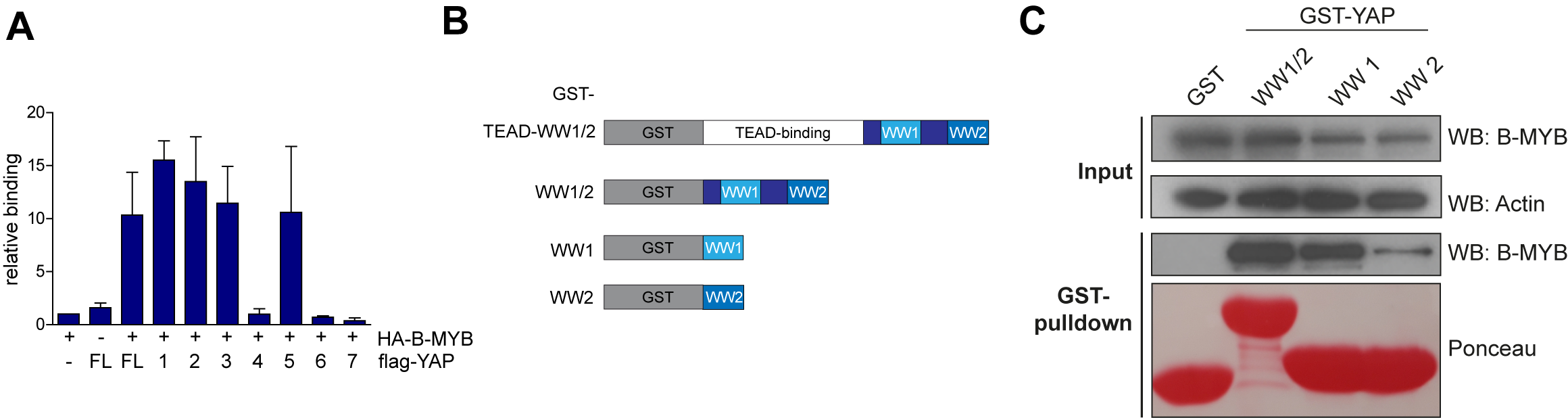

Supplement: S5 Fig — A) Densiometric quantification of binding data shown in Fig 6B using ImageJ. Binding is relative to HA-B-MYB control cells. n = 3 biological replicates. B) Scheme of the GST fusion constructs used in pulldown experiments in Fig 6D and S5C Fig C) Pulldown experiments of the indicated GST fusion proteins with HA-B-MYB. Bound B-MYB was detected by immunoblotting with an HA-antibody. Input: 3% of the lysate used for the pulldown was loaded onto the gel. Actin served as a control. Ponceau staining was used to detect the recombinant GST-proteins. (TIF) [file pgen.1008818.s005.tif]

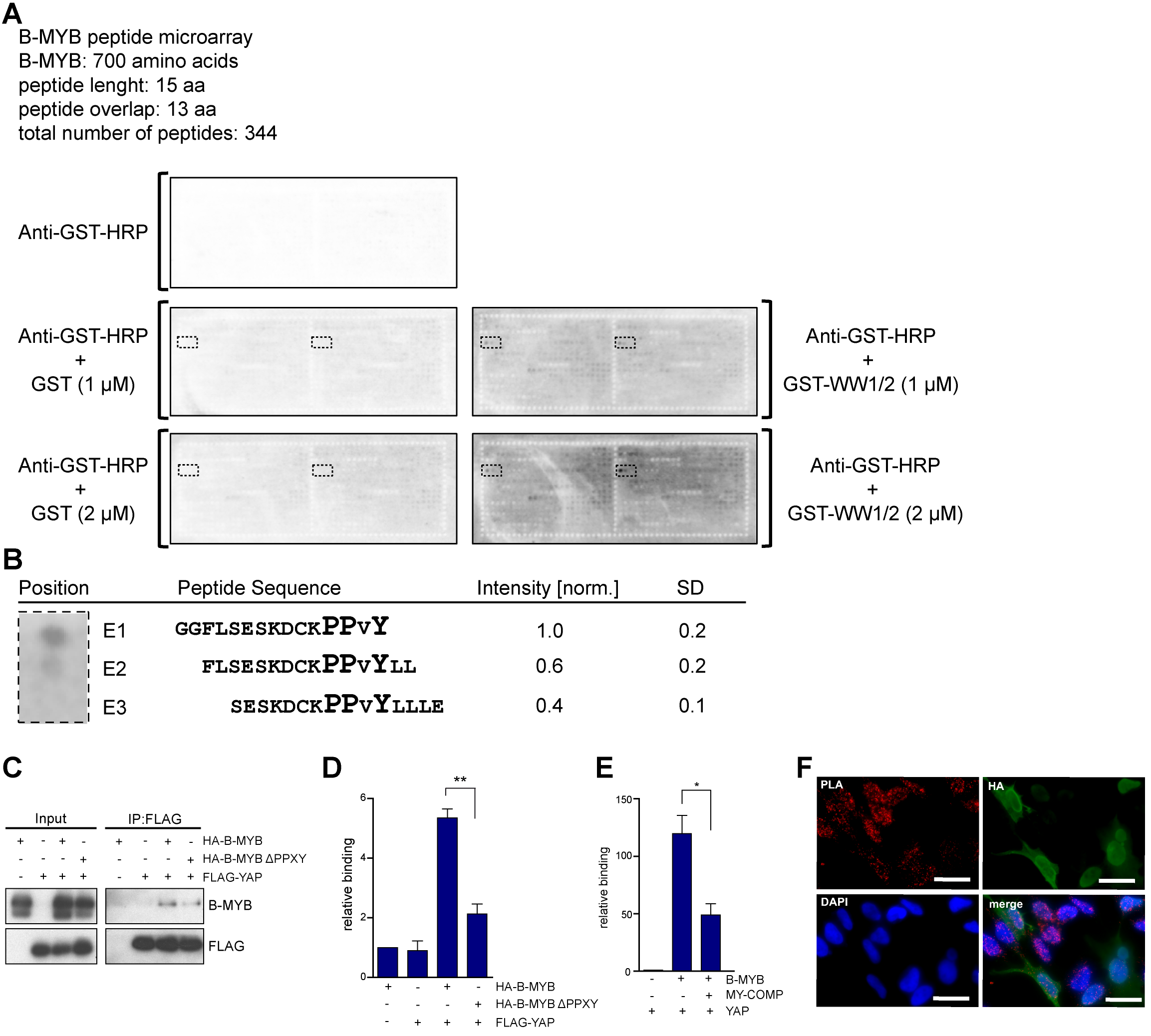

Supplement: S6 Fig — A) μSPOT based mapping of YAP WW1/2 interactions. An overlapping peptide library to display the whole B-MYB protein was probed with only Anti-GST-HRP, with purified recombinant GST and Anti-GST-HRP (control) or with purified recombinant GST-WW1/2 and Anti-GST-HRP. Binding was detected by chemiluminescene. Most prominent binding of YAP is observed at position E1. The respective B-MYB derived peptide contains a WW-binding PPXY motif. B) Zoomed view of marked region in A showing spots corresponding to the most prominent binding sequences within B-MYB with relative binding intensities and standard deviation (SD). C) Co-immunoprecipitation experiments of the indicated B-MYB constructs with flag-YAP. Lysates of HeLa cells expressing flag-YAP and HA-B-MYB (wt and ΔPPXY) were immunoprecipitated with flag-antiserum and immunoblotted with an anti-HA-antibody and anti-flag antibody. 3% percent of total lysate was immunoblotted (Input). D) Densiometric quantification of the binding of HA-MYB and HA-B-MYBΔPPXY to flag-YAP using Image J. Binding is relative to HA-B-MYB control cells. E) Densiometric quantification of the binding of HA-B-MYB to flag-YAP in presence and absence of MY-COMP using Image J. Binding is relative to FLAG-YAP control cells. N = 3 biological replicates. F) Proximity ligation assay (PLA) of endogenous YAP and B-MYB upon transfection of MY-COMP. Cell expressing MY-COMP were identified by HA staining (green). Example microphotographs of the experiments shown in Fig 7F. D), E): Error bars indicate SEMs. Student’s t-test. * = p<0.05, ** = p<0.01. (TIF) [file pgen.1008818.s006.tif]

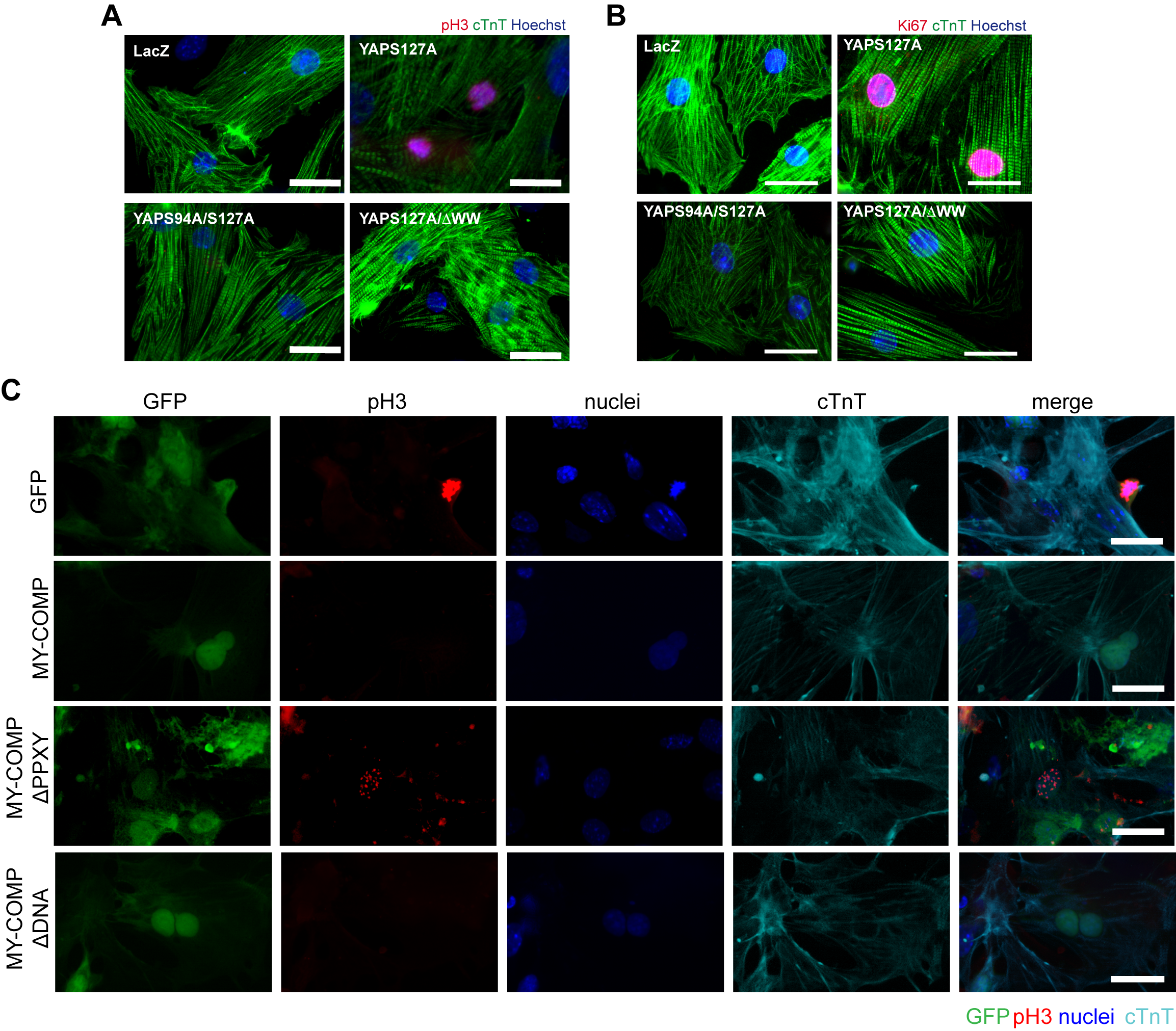

Supplement: S7 Fig — A) and B) Cardiomyocytes were transduced with Ade-LacZ, Ade-YAP[S127A], and Ade-YAP[S127A/S94A] or with Ade-YAP[S127A/ΔWW]. The fraction of pH3-positive positive (A) and Ki67 (B) cardiomyocytes was determined by immunostaining. Example microphotographs of the experiment shown in Fig 6E and 6F. Scale bar: 25 μm. C) Embryonal cardiomyocytes were infected with adenoviruses expressing GFP, MY-COMP, with MY-COMP,ΔPPXY or with MY-COMP,ΔDNA each coupled to GFP through a T2A self-cleaving peptide. Mitotic cells were quantified by staining for phospho-H3 (red). Scale bar: 25 μm. (TIF) [file pgen.1008818.s007.tif]

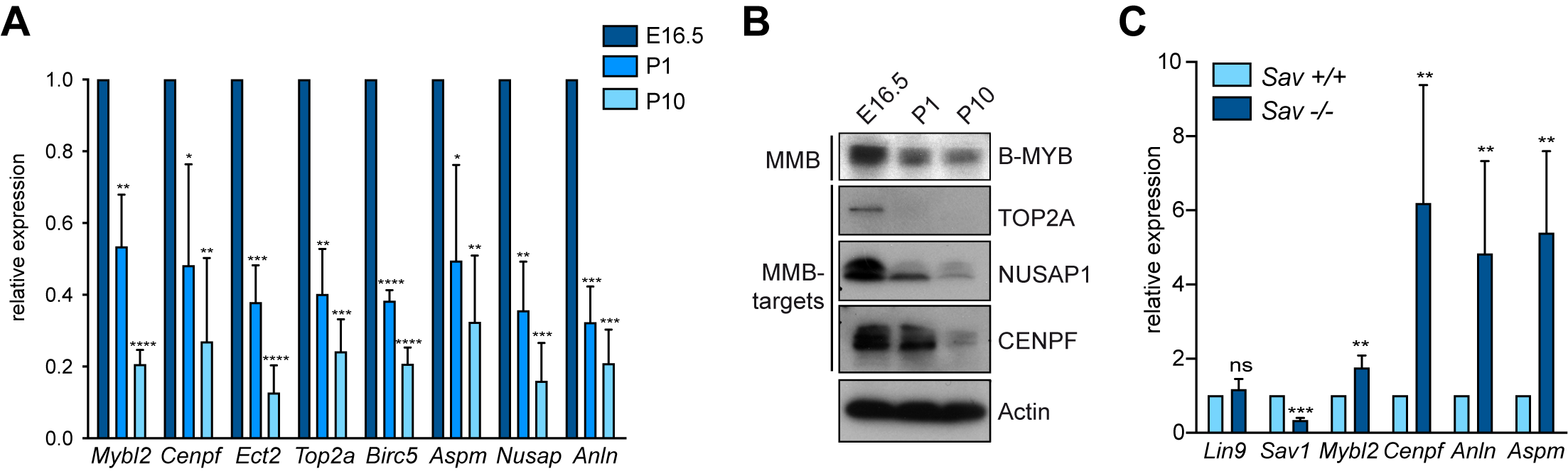

Supplement: S8 Fig — A) The expression of the indicated MMB target genes relative to Hprt and Actin was investigated in E16.5, P1 and P10 hearts. n = 3 independent replicates. B) The expression of the indicated proteins in lysates prepared from hearts at the different developmental stages was investigated by immunoblotting. β-Actin served as a control. C) Expression of the indicated genes in Nkx2.5-Cre; Sav1+/+ and Nkx2.5-Cre; Sav1fl/fl in hearts of P1 mice was investigated. n = 7 independent Nkx2.5-Cre; Sav1+/+ and Nkx2.5-Cre; Sav1fl/fl animals. (TIF) [file pgen.1008818.s008.tif]

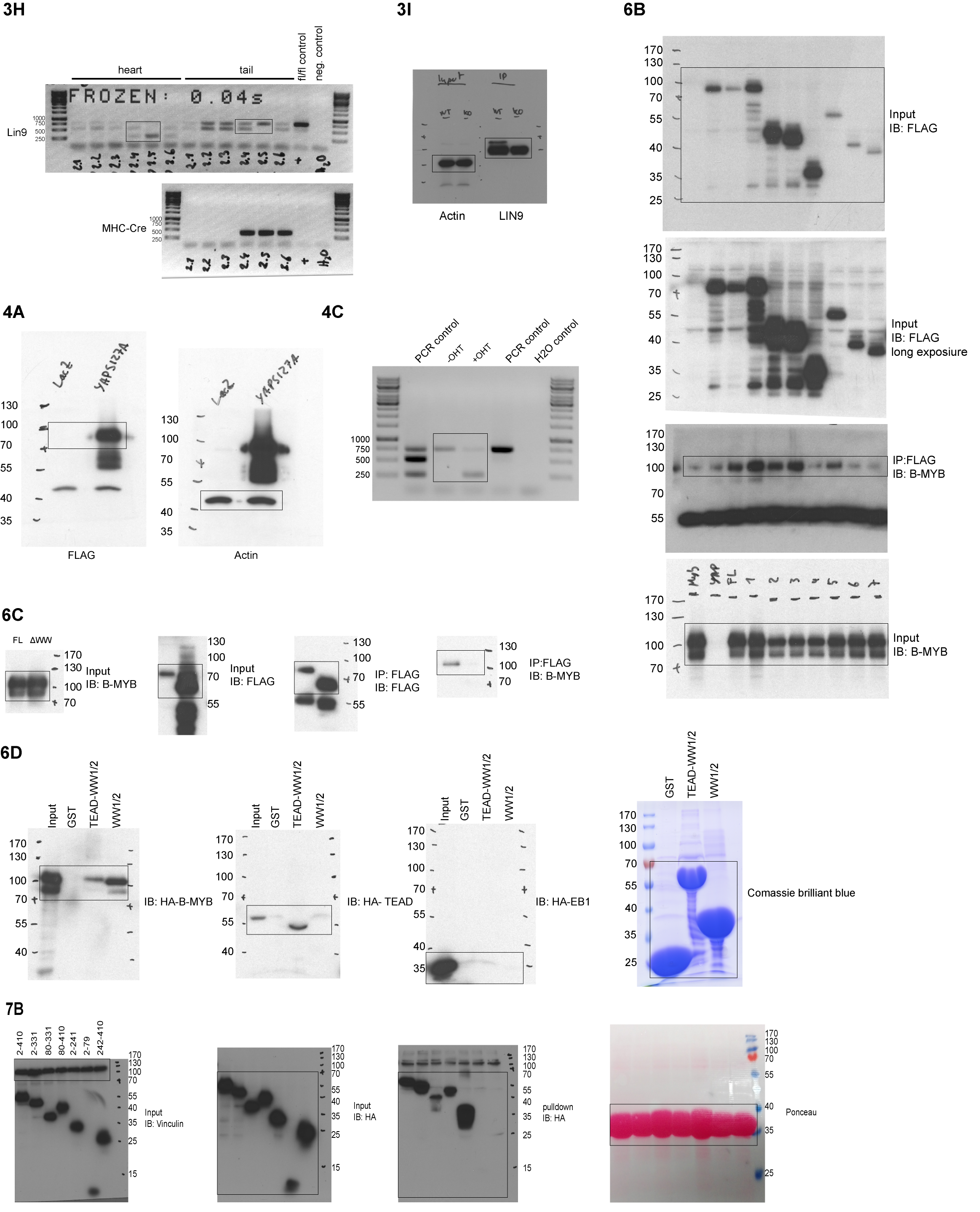

Supplement: S9 Fig — Part 1. (TIF) [file pgen.1008818.s009.tif]

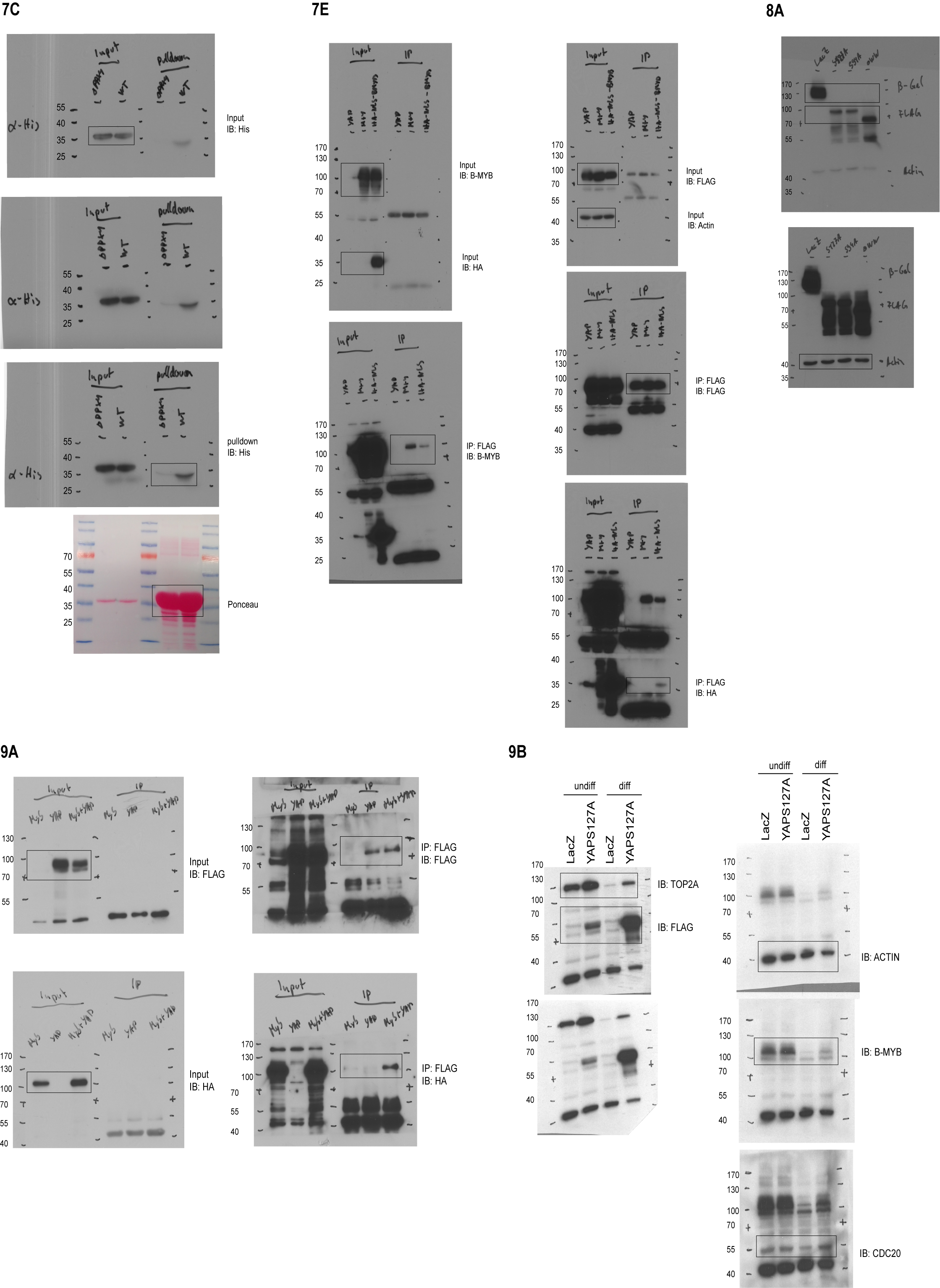

Supplement: S10 Fig — Part 2. (TIF) [file pgen.1008818.s010.tif]

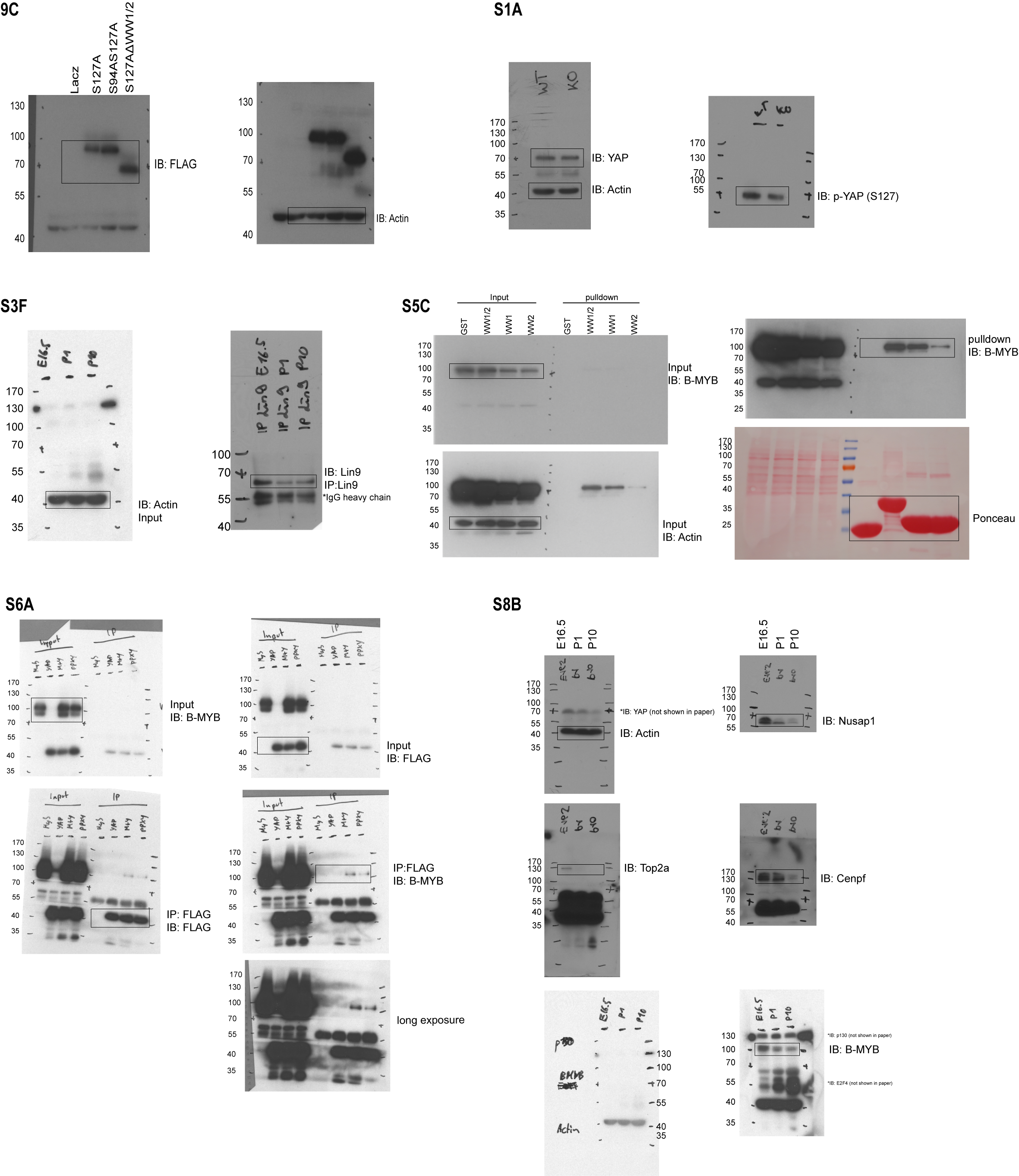

Supplement: S11 Fig — Part 3. (TIF) [file pgen.1008818.s011.tif]
